# A case report of Pallister-Killian syndrome with an unusual mosaic supernumerary marker chromosome 12 with interstitial 12p13.1-p12.1 duplication

**DOI:** 10.3389/fgene.2024.1331066

**Published:** 2024-03-11

**Authors:** T. V. Karamysheva, I. N. Lebedev, L. I. Minaycheva, L. P. Nazarenko, A. A. Kashevarova, D. A. Fedotov, N. A. Skryabin, M. E. Lopatkina, A. D. Cheremnykh, E. A. Fonova, T. V. Nikitina, E. A. Sazhenova, M. M. Skleimova, N. A. Kolesnikov, G. V. Drozdov, Y. S. Yakovleva, G. N. Seitova, K. E. Orishchenko, N. B. Rubtsov

**Affiliations:** ^1^ Institute of Cytology and Genetics, Siberian Branch of Russian Academy of Sciences (SB RAS), Novosibirsk, Russia; ^2^ Department of Genetic Technologies, Novosibirsk State University, Novosibirsk, Russia; ^3^ Research Institute of Medical Genetics, Tomsk National Research Medical Center of the Russian Academy of Sciences, Tomsk, Russia; ^4^ Department of Medical Genetics, Siberian State Medical University, Tomsk, Russia

**Keywords:** Pallister-Killian syndrome (PKS), mosaic trisomy 12p, a supernumerary marker of chromosome 12, the chromosomal region 12pter-12q11, chromosomal abnormalities, duplication 12p13.33-p11.1

## Abstract

Pallister-Killian syndrome (PKS) is a rare inherited disease with multiple congenital anomalies, profound intellectual disability, and the presence in the karyotype of sSMC - i(12)(p10). The frequency of PKS may be underestimated due to problems with cytogenetic diagnosis caused by tissue-specific mosaicism and usually a low percentage of peripheral blood cells containing sSMC. Such tissue-specific mosaicism also complicates a detailed analysis of the sSMC, which, along with the assessment of mosaicism in different tissues, is an important part of cytogenetic diagnosis in PKS. Unfortunately, a full-fledged diagnosis in PKS is either practically impossible or complicated. On the one hand, this is due to problems with the biopsy of various tissues (skin biopsy with fibroblast culture is most often used in practice); on the other - a low percentage of dividing peripheral blood cells containing sSMC, which often significantly complicates the analysis of its composition and organization. In the present study, a detailed analysis of sSMC was carried out in a patient with a characteristic clinical picture of PKS. A relatively high percentage of peripheral blood cells with sSMC (50%) made it possible to perform a detailed molecular cytogenetic analysis of *de novo* sSMC using chromosomal *in situ* suppression hybridization (CISS-hybridization), multicolor FISH (mFISH), multicolor chromosome banding (MCB), array CGH (aCGH), and quantitative real-time PCR (qPCR), and short tandem repeat (STR) - analysis. As a result, it was found that the sSMC is not a typical PKS derivative of chromosome 12. In contrast to the classical i(12)(p10) for PKS, the patient’s cells contained an acrocentric chromosome consisting of 12p material. Clusters of telomeric repeats were found at the both ends of the sSMC. Furthemore, the results of aCGH and qPCR indicate the presence of interstitial 8.9 Mb duplication at 12p13.1-p12.1 within the sSMC, which leads to different representations of DNA from different segments of 12p within cells containing sSMC. The obtained data raise the question of the instability of the sSMC and, as a consequence, the possible presence of additional rearrangements, which, in traditional cytogenetic analysis of patients with PKS, are usually described as i(12)(p10).

## 1 Introduction

Small supernumerary marker chromosomes (sSMCs) are additional centric chromosomal fragments of small size that cannot be precisely characterised by classical cytogenetic methods based on staining of trypsin-treated chromosomes (GTG-banding). They are known to be primarily not larger than chromosome 20 (Liehr et al., 2004a; Shaffer et al., 2013). The largest sSMCs by the presence of euchromatin in them are the sSMCs representing inverted duplications of the short arms of chromosomes 12 and 18. The first sSMCs were described in 1961 by Ilberry. According to modern estimates, they are present in the karyotypes of about 3 million people (Liehr et al., 2004b; Liehr, 2014). Most sSMCs are non-pathogenic and not associated with developmental disorders. There are examples of familial sSMCs, which were transmitted through several generations. It was assumed that such chromosomes consisted of heterochromatin and did not contain transcriptionally active genes (Karamysheva et al., 2020). It was later shown that sSMCs, which include euchromatin regions up to 5 Mb in size, are also usually non-pathogenic (Liehr, 2014). Moreover, a case of a ring non-pathogenic sSMC with euchromatin regions with more than 9 Mb in size was described (Lebedev et al., 2021). It was suggested that the euchromatin regions of such chromosomes are located in the interphase nucleus compartment, which mainly contains inactive chromatin, and the transcriptional activity of the genes of the euchromatin regions of such sSMCs is largely or entirely suppressed (Nichols and Corces, 2021). Nevertheless, there is a clear trend that the larger the euchromatin region that an sSMC contains, the stronger the pathogenic effect associated with its presence. Approximately in 30% of cases, carriers of sSMCs have an abnormal phenotype (Liehr and Al-Rikabi, 2019). The potential clinical significance of sSMCs has stimulated the development of methods for determining the origin of the sSMC and its structure (Douet-Guilbert et al., 2007). These methods include MCB (Chudoba et al., 1999), AcroM-FISH (Langer et al., 2001), SubcenM-FISH (Starke et al., 2003) and CenM-FISH (Nietzel et al., 2001). The creation of microdissection DNA probes, prepared from sSMCs, followed by FISH with metaphase chromosomes or sequencing of microdissected DNA libraries, prepared from sSMCs, has proven highly effective (Liehr T., 2012; Lebedev et al., 2021). Sometimes, the description of sSMCs is complicated by possible rearrangements within the sSMCs. Also, mosaic marker chromosomes, found in only some of the cells in the patients, may present a separate type of sSMC. Their study and diagnosis requires the development of specific approaches.

One of the most striking cases of sSMC mosaicism is associated with Pallister-Killian syndrome (PKS). It is usually associated with tetrasomy 12p (mosaic isochromosome syndrome invdup(12p)) (OMIM# 601803). PKS is a multiple congenital anomaly syndrome associated with intellectual disability. While the cases of PKS are rare, the frequency of sSMCs, which is invdup(12p), may be significantly higher at early stages of embryo development, but early neonatal death of carriers of invdup(12p) can lead to an underestimation of its frequency. Possibly, the death of newborns with congenital diaphragmatic hernia (this developmental defect is specific and frequent in PKS) may be, at least in part, associated with the presence of invdup(12p) in some cells of these newborns (Kostyk and Salik, 2022). It should be noted that detecting such an sSMC in these cases may be complicated by tissue mosaicism and a low frequency of sSMC occurrence in the cells of tissues usually used for diagnosis. Unfortunately, at present, there is only limited information about the presence of invdup(12p) in cells of most different tissues of patients with PKS (Dufke et al., 2001), which complicates not only the cytogenetic diagnostics but also the development of reliable protocols for its performing.

The present study describes an unusual case where a patient with PKS has an sSMC in a large percentage of peripheral blood cells and represents an acrocentric chromosome with interstitial duplication. To describe sSMC, such methods as GTG-differential chromosome staining (G-bands after trypsin and Giemsa), array-based comparative genomic hybridization (aCGH), multicolor fluorescence *in situ* hybridization (mFISH), FISH locus-specific probes and painting probes specific to chromosomal arms and individual regions of chromosome 12 were used. Quantitative real-time PCR was also performed to assess the representation in blood cells of specific loci from the short arm of chromosome 12. STR analysis was used to study the origin and inheritance of the sSMC(12).

## 2 Materials and methods

### 2.1 The clinical case

**FIGURE 1:**
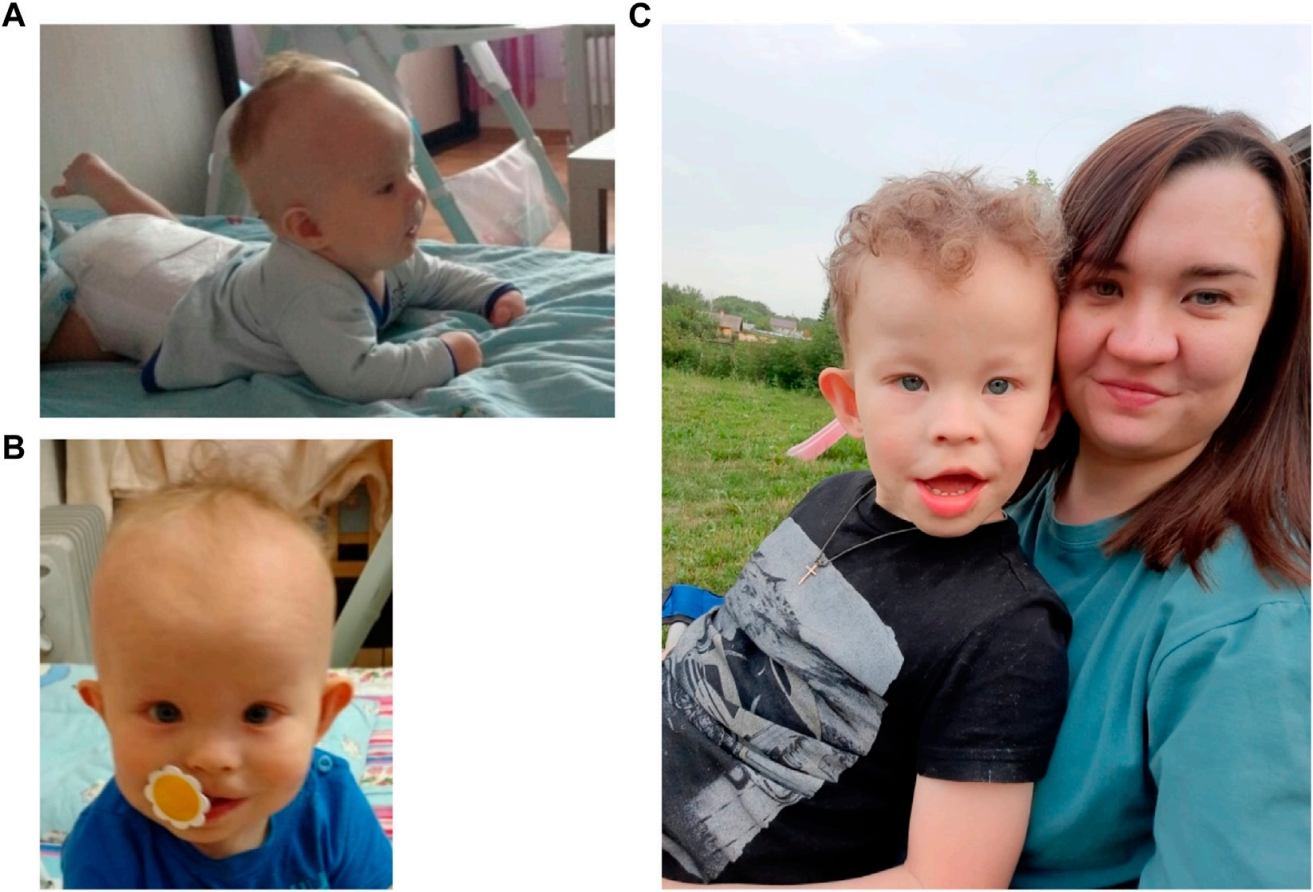
The patient at the ages of 4 months **(A)**, 1 year and 9 months **(B)** and 6 years with mother **(C)**.

A family with a child sought consultation from a medical geneticist. The patient was 1 year and 9 months old (Figure 1), height was 84 cm (<50^th^ centile), and body weight was 13 kg (75^th^ centile). The child was the product of the first pregnancy, delivered by term caesarian section (birth weight–4,200 g (90^th^ centile, length–57 cm (>97^th^ centile)). A congenital defect—polydactyly of the right foot—was prenatally detected during the second-trimester ultrasound fetal examination. The family declined invasive diagnostic procedures intended to rule out chromosomal abnormalities. At birth, the child had preaxial polydactyly and syndactyly of the II, III, and IV toes of both feet and syndactyly of the II, and III fingers of both hands, with facial dysmorphism. The child’s neurological and motor development was delayed: started sitting at 1 year, crawling at 1 year 6 months, walking at 1 year and 7 months. The child was under the supervision of a local neurologist with a diagnosis of “perinatal encephalopathy, hypertensive syndrome, mild tetraparesis, delayed psychomotor development".

On examination of the patient, carried out as part of the present study, the following developmental defects and facial dysmorphisms were identified: macrocephaly, a protruding forehead with a high hairline, absence of hair in the frontal-temporal area, a flat face, hypertelorism, upslanted palpebral fissures, epicanthus, a wide and flat nose bridge, short nose with anteverted nostrils, a long philtrum, a thin upper lip, micrognathia, large protruding ears, a short neck, wide hands, and feet, transverse palmar crease, preaxial polydactyly and syndactyly of the II, III, IV toes of both feet, syndactyly of the II, III fingers of both hands. The body was proportionate; there was no limb shortening, skin color was normal, hypo- and hyperpigmentation were absent. The child had a pronounced intellectual disability. Delayed teething from 1 year and 6 months was noted. The patient had no internal organ developmental defects. A magnetic resonance imaging scan of the brain was performed and revealed the following signs: decreased volume of the brain’s white matter; multiple focal and periventricular leukopathy, hypoplasia of the corpus callosum; moderate expansion of external and internal replacement spaces.

At the time of examination 6-year-old patient (Figure 1C) was 114 cm tall (50^th^ centile) and weighed 22 kg (50-75^th^ centile). Hydrocephalus, high forehead, frontal bossing, prominent occiput, epicanthus, wide nose bridge, diastema, dysplastic protruding ears, short neck, nipple hypertelorism, and wide umbilical ring were noted. The patient exhibited restlessness, moodiness (often screamed), chaotic and stereotypical hand movements (shaking toys). He did not respond to his name if called by a stranger. There was no fear of strangers or of the road with moving vehicles. Speech was represented by inadequate vocalisms and very rarely by individual words. There was understanding of speech, but due to behavioral characteristics it was very selective. There was auto-aggressive behavior (chewed his hands). Self-care skills were not sufficiently developed (did not ask to go to the toilet). A psychiatrist’s diagnosis at the age of 6 years was childhood autism caused by organic brain damage, combined with intellectual disability.

On examination of the patient’s mother, a flat face, large protruding ears (otoplasty performed), hypertelorism, upslanted palpebral fissures, epicanthus, wide flat nose bridge, a long philtrum, a thin upper lip, syndactyly of the III and IV fingers of the left hand (surgery), umbilical hernia, bilateral inguinal hernia (surgery) were noted. Her intellect is normal. The patient’s maternal uncle has syndactyly of the III and IV fingers of the left hand (reported, not examined). There was no intellectual disability. The patient’s maternal grandfather had preaxial polydactyly of the feet, syndactyly of I and II toes (reported, not examined). There was no intellectual disability. Parents deny consanguinity. No other cases of congenital developmental defects or intellectual disability were identified in the pedigree (Supplementary Figure S1).

### 2.2 Experimental design

The analysis of chromosomal abnormality included several steps: (1) GTG-banding (G-bands after trypsin and Giemsa) (Seabright, 1971); (2) 24-color FISH for human chromosome identification; (3) Generation of microdissected DNA probe followed by FISH with metaphase chromosomes of patient and healthy donor (Rubtsov et al., 2000); (4) FISH analysis with various DNA probes, Whole Chromosome Paint prepared from chromosome 12 (WCP12), Partial Chromosome Paints prepared from regions of chromosome 12; (5) Multicolor Banding of chromosome 12 (Chudoba et al., 1999); (6) array-based comparative genomic hybridization (aCGH) (Nikitina et al., 2021); (7) Multicolor fluorescence *in situ* hybridization (mFISH) (Liehr and Claussen, 2002a); (8) analysis of locus copy number variation with quantitative real-time PCR (Untergasser et al., 2012); (9) STR-analysis for determination of parental origin and inheritance of supernumerary marker chromosome 12 (Fan and Chu, 2007).

### 2.3 Metaphase chromosome preparation and GTG-Banding analysis

Conventional cytogenetic analysis was performed with GTG-banded metaphases of peripheral blood lymphocytes from the patient and his parents at a 500-band resolution. Lymphocytes from peripheral blood were cultivated according to the standard protocol (Verma and Babu, 1998). Slides with metaphase and prometaphase chromosomes for FISH were prepared as previously described (Henegariu et al., 2001). GTG banding of metaphase chromosomes was performed according to the standard protocol. Banded chromosomes were captured and analyzed on light microscope Axio Scope. A1 (Carl Zeiss, Jena, Germany) equipped with CoolCube 1 CCD camera (MetaSystems, Germany) and using Ikaros Karyotyping Systems software V5.9.1. (MetaSystems, Altlussheim, Germany).

### 2.4 24XCyte - Human Multicolor FISH probe

Multicolor fluorescence *in situ* hybridization (mFISH) was performed using the 24XCyte color kit for human chromosomes (MetaSystems, Altlussheim, Germany) following the supplier’s recommendations.

### 2.5 Generation of WCP12, chromosome arm specific paints and partial chromosome paints by metaphase chromosome microdissection followed with DOP-PCR

Microdissected DNA libraries were generated from DNA of 5–10 copies of respective chromosome or chromosomal regions followed with standard treatment and DOP-PCR as described earlier (Rubtsov et al., 2000; Karamysheva et al., 2001). The DNA labeling of microdissected DNA libraries was performed according to standard protocol (Liehr et al., 2002b) with AlexaFluor488-5-dUTP (Molecular Probes, Eugene, OH, United States of America) or TAMRA-5-dUTP (5-Tetramethylrhodamine-dUTP, Molecular Probes, Eugene, OH, United States) (Telenius et al., 1992). As a result the following WCP and PCPs were prepared: WCP12 derived from the entire chromosome 12; PCP12p and PCP12q derived from the short- and long-arms of chromosome 12, correspondently; PCP12-1 and PCP12-2 from proximal and distal regions of the 12p. The quality of all prepared microdissected DNA probes were tested by Chromosomal *in situ* Suppression hybridization (CISS-Hybridization) on metaphase chromosomes of a donor with normal karyotype.

### 2.6 CISS-hybridization of microdissected DNA probes

CISS-hybridization of microdissected DNA probes on metaphase chromosomes of a healthy donor or patient was performed according to a standard protocol of CISS-Hybridization with suppression of hybridization of repetitive DNA (Lichter et al., 1988). The chromosome staining was performed with 4’,6-Diamidino-2-phenylindole dihydrochloride (DAPI) (Sigma, Darmstadt, Germany). Chromosomes and chromosomal regions were identified by analysis of inverted DAPI banding and were described according to the International System for Human Cytogenomic Nomenclature 2020 (McGowan-Jordan et al., 2020).

### 2.7 Multicolor chromosome banding (MCB)

Additional description of chromosome 12 regions based of their DNA content was performed with MCB (Chudoba et al., 1999; Liehr and Claussen, 2002a) with six PCPs generated from 12pter-12p12 (PCP12-1), 12p12-p11 (PCP12-2), 12q12–q14 (PCP12-3), and 12q14–q21(PCP12-4) 12q21–q23 (PCP12-5), and 12q23–q24(PCP12-6) chromosomal regions (Liehr et al., 2002b). PCPs were prepared from microdissected DNA libraries by labeling with TAMRA-dUTP (PCP12-1), AlexaFluor488-5-dUTP (PCP12-2), DEAC-dUTP (PCP12-3), TAMRA-dUTP (PCP12-4), AlexaFluor 488-dUTP (PCP12-5) or Texas Red™-12-dUTP (PCP12-6), CISS-Hybridization with the PCPs was carried out according to standard protocol (Chudoba et al., 1999; Karamysheva et al., 2022).

### 2.8 The microscopy and image analysis

Microscopic analysis after FISH was performed on AxioPlan 2 *Imaging* microscope (Zeiss, Germany) with the filters kit #49 (Zeiss, Germany), SP101 FITC (CHROMA, Wixom, MI, United States), and SP103v1 Cy3tmv1 (CHROMA, Wixom, MI, United States), Set 01(FT 395, LP 397, BP 365/12) (Zeiss, Germany), Set 32 (BP 665/45, FT 695, BP 725/50), (Zeiss, Germany), CCD camera (CV M300, JAI Corporation, Miyazaki, Japan). For signal registration, ISIS5 software (MetaSystems GmbH, Germany) was used. This microscopy was performed at the Core Facility for microscopic analysis of biological objects at the Institute of Cytology and Genetics SB RAS, Russia. For image analysis and generation of the Multicolor-Chromosome Banding ISIS5 software (MetaSystems GmbH, Germany) was applied.

### 2.9 aCGH

Array-based comparative genomic hybridization (aCGH) for proband and his mother was performed using SurePrint G3 Human CGH 8 × 60K microarrays (Agilent Technologies, Santa Clara, CA, United States) with 41 kb overall median probe spacing according to the manufacturer’s recommendations. Labelling and hybridization of the patient’s, maternal and reference DNA (#5190-3,797, Human Reference DNA Female, Agilent Technologies, Santa Clara, CA, United States) were performed using enzymatic labelling and hybridization protocols, v.7.5 (Agilent Technologies, Santa Clara, CA, United States). Array images were acquired with an Agilent Sure Scan Microarray Scanner (Agilent Technologies, Santa Clara, CA, United States). Data analysis was performed using CytoGenomics Software, v.5.1.2.1 (Agilent Technologies, Santa Clara, CA, United States) and the publicly available Database of Genomic Variants (DGV) resources. Human genome assembly 19 (hg19) were used to describe the molecular karyotype revealed by aCGH according to ISCN 2020 (McGowan-Jordan et al., 2020).

DNA sample from the control spontaneous abortion specimen with trisomy 12 were labeled by SureTag Labeling Kit (#G9502A, Agilent, United States) together with the control female DNA sample. Purification was done by SureTag Purification Columns (#5190-7,730, Agilent, United States). Labeled DNA samples were hybridized using GenetiSure Pre-Screen microarray (8 × 60K) (#G5963A, Agilent, United States) at 67°C for 24 h according to manufacturer’s protocol. Microarray images were obtained using SureScan Microarray Scanner (Agilent, United States) and analyzed by Agilent Feature Extraction (v.10.7.3.1) and CytoGenomics software (v.5.3).

### 2.10 Analysis of locus copy number variation with quantitative real-time PCR

Specific amplification primers for quantitative real-time PCR analysis of copy number variations within 12p and 12q were selected using Primer 3 software (Supplementary Table S1). The presence of mosaicism for dup12p13.33-p11.1 and trip12p13.1-p12.1 and the normal copy number of 12q was tested using genomic DNA from peripheral blood lymphocytes from the patient, positive control #1 (DNA from spontaneous abortion with trisomy 12) and positive control #2 (DNA from the same spontaneous abortion with trisomy 12 two times diluted with control genomic DNA with balanced karyotype (Agilent Technologies)) using the AriaMx Real-Time PCR System (Agilent Technologies). Parental DNA was used to test the origin of the sSMC(12). The control gene was *HEXB*, which encodes the β subunit of hexosaminidase and is located at 5q13 (Supplementary Table S1). Real-time PCR was performed using 25 ng of DNA (10 ng/μL), 2.5 μL (1 μM/L) of forward and reverse primers, 12.5 μL of 2× BioMaster HS-qPCR SYBR Blue (BioLabMix, Novosibirsk, Russia), and RNase-free water to 20 μL (per one well). The real-time PCR conditions were as follows: initial incubation at 95°C for 10 min followed by 40 cycles of 15 s at 95°C, 30 s at 60°C, and 30 s at 72°C. Three technical replicates were run for each sample. The obtained values of C_T_ for test and reference (control) DNA amplification with primers for test and reference genes were analyzed using the following: average value for C_T_, logQT test primer = (Ct test DNA − CT reference DNA)/slope, logQT test primer − (Ct test DNA − Ct reference DNA)/slope, (logQT test primer − log QT control primer), and fold change = 10^logQT test primer − logQT control primer^. Fold change values were used to build chart.

### 2.11 Short tandem repeat (STR)-analysis

Analysis of short tandem repeats (STR) was carried out using the “COrDIS Expert 26” kit for DNA identification of 26 STR markers (Gordis, Russia) and specially designed markers for 12q21.32 and 12q23.31 regions. The analysis using the “COrDIS Expert 26” kit included identification of 26 loci: D1S1656 (1q42); D2S441 (2p14); D3S1358 (3p21.31); D5S818 (5q23.2); D7S820 (7q21.11); D8S1179 (8q24.13); D10S1248 (10q26.3); D12S391 (12p13.2); D13S317 (13q31.1); D16S539 (16q24.1); D18S51 (18q21.33); D21S11 (21q21.1); D22S1045 (22q12.3); CSF1PO (5q33.1); FGA (4q31.3); SE33 (6q14); TH01 (11p15.5); TPOX (2p25.3); VWA (12p13.31); D6S1043 (6q15); D19S433 (19q12); D2S1338 (2q35); DYS391 (Y); Аmel X (Xp22.1-22.3); Аmel Y (Yp11.2); SRY (Yp11.2); Yindel (Yq11.221).

To analyze the origin of chromosome 12, the following STR loci were used: for the region 12p13.31–VWA; 12p13.2 - D12S391; 12q23.31 - D12ATA63; and for region 12q21.32 (gene *CEP290*) - 12q_8658, 12q_8754, 12q_8771, 12q_8795.6, 12q_8860 (Supplementary Table S2).

The PCR products of individual loci were separated on a NANOFOR-05 genetic analyzer (Synthol, Russia) in the presence of DNA length standards S550 (CORDIS) under the conditions recommended by the manufacturer. Fragment-size analysis was performed using GeneMarker Software (V.3.0.1) (State College, PA, United States).

### 2.12 Ethics

The study was carried out according to the principles of the Declaration of Helsinki. Informed consent for cytogenetic investigations was obtained from the parents. The study was approved by the local Ethics Committee of the Research Institute of Medical Genetics, Tomsk National Research Medical Center of the Russian Academy of Sciences (# 15 from 28 February 2023).

## 3 Results

### The primary cytogenetic analysis

**FIGURE 2:**
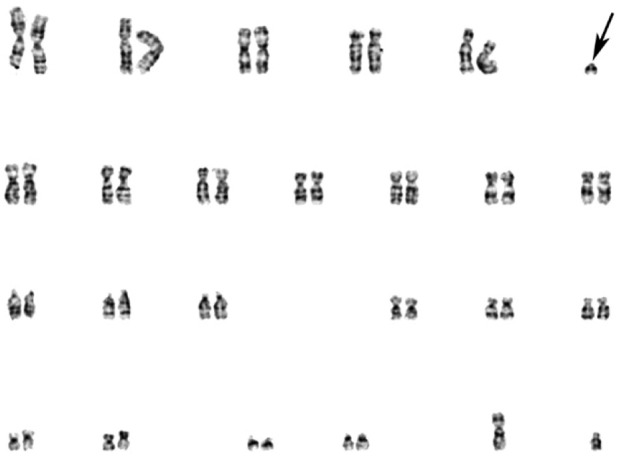
GTG-banded patient’s chromosomes. Arrow points to the sSMC.

The GTG-banding of chromosomes from 20 metaphase plates of PHA-stimulated peripheral blood cells revealed an sSMC in 10 of them, allowing us to describe the karyotype as 47,XY,+mar[10]/46,XY[10] (Figure 2).

**FIGURE 3:**
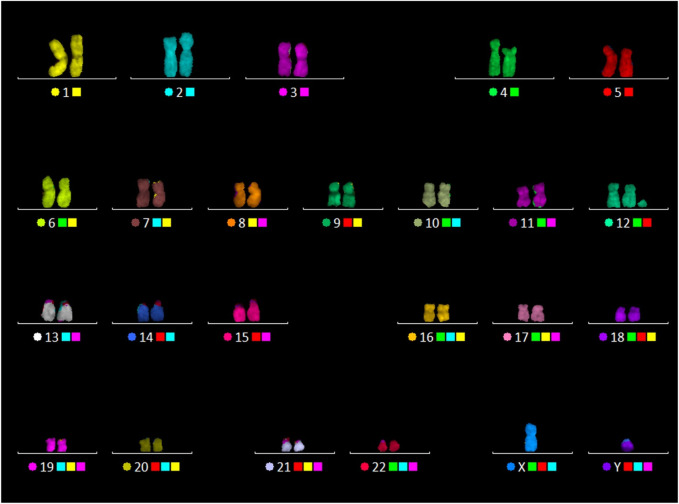
24-color FISH with the patient’s metaphase chromosomes.

A 24-color FISH with a DNA probe “24XCyte Human Multicolor FISH (mFISH) Probe Kit” (MetaSystems, Germany) showed that the sSMC consists of chromosome 12 material (Figure 3).

To confirm the results of 24-color FISH and clarify the composition of the sSMC, the painting was performed using WCP12, PCP12p, and PCP12q. CISS-hybridization with WCP12 painted chromosome 12 in all metaphase plates and in the presence of sSMC (47,XY,+mar), also the marker chromosome (Supplementary Figure S2). CISS-hybridization with PCP12p painted the short arm of chromosome 12 and, in the presence of sSMC (47,XY,+mar), also the marker chromosome. PCP12q in all metaphase plates painted only the long arm of chromosome 12 (Supplementary Figure S3). There was no signal on other chromosomes.

Thus, the obtained results unequivocally indicate that the sSMC consists of material derived from the short arm of chromosome 12. For a more accurate description of the sSMC, images of it and sSMC multicolor banding were obtained using region-specific DNA probes from chromosome 12.

**FIGURE 4:**
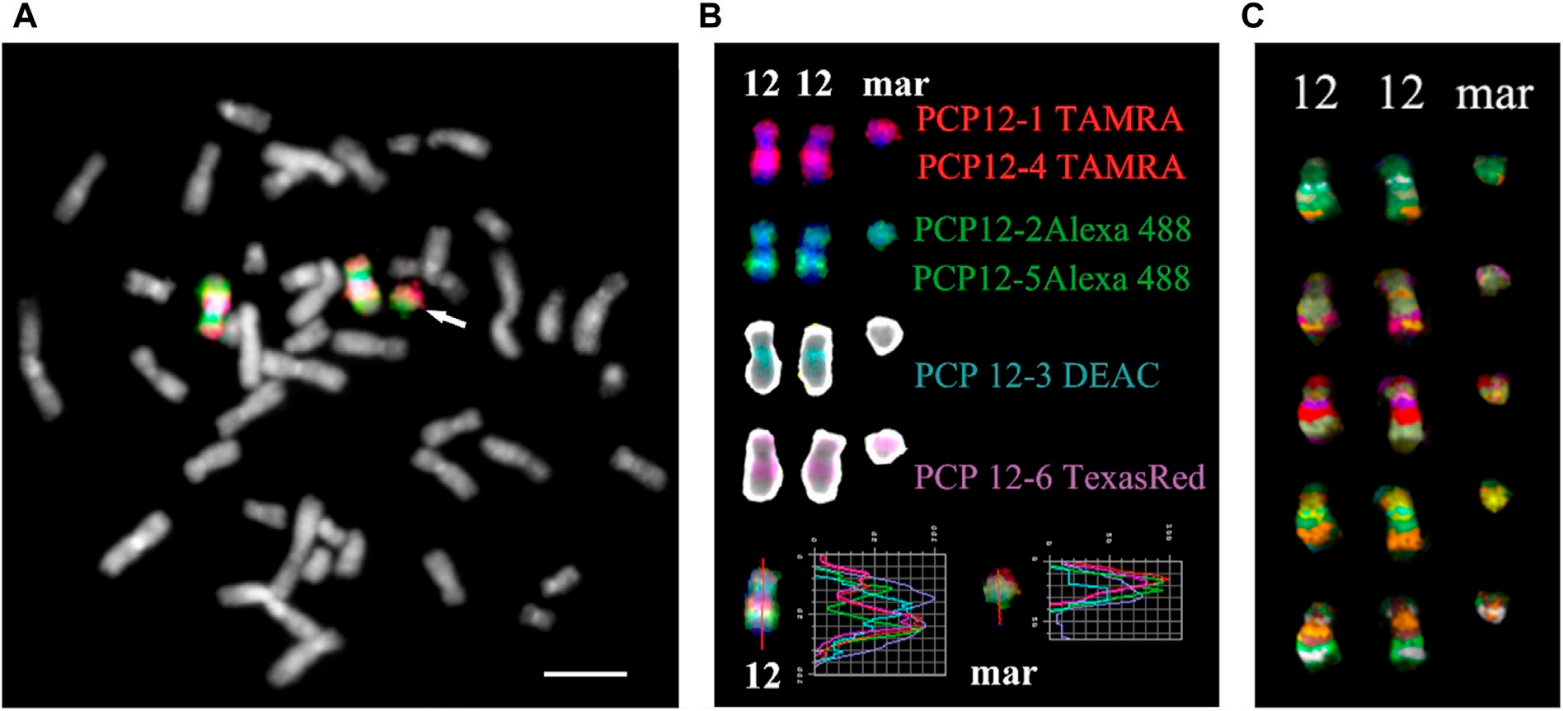
Result of high-resolution multicolor banding (MCB) after application of a chromosome 12 specific probe cocktail. **(A)** The resulting pseudocolor MCB patterns for the two normal chromosomes 12 and the derivative chromosome 12 (mar) of the presented PKS patient. The arrow points to the small supernumerary marker chromosome 12. **(B)** The localization of the chromosome 12 region-specific partial chromosome paints and the used fluorochromes are shown. At the bottom are the intensity profiles. The red line on chromosome 12 and the marker is the line along which the intensity profiles were measured. **(C)** MCB of a chromosome 12 and marker are presented. Scale bar 50 µm.

For obtaining multicolor banding, simultaneous FISH of six microdissected DNA probes (PCP12-1-TAMRA, PCP12-2-Alexa488, PCP12-3-DEAC, PCP12-4-TAMRA, PCP12-5-Alexa488, and PCP12-6-TexasRed), labeled with different fluorochromes, was performed on the patient’s metaphase chromosomes (Chudoba et al., 1999). The intensity profiles of the FISH signal along chromosome 12 and the MCB images obtained using MetaSystems Software (MetaSystems Hard & Software GmbH, Altlussheim, Germany) (Chudoba et al., 1999; Liehr et al., 2002c; Karamysheva et al., 2022) are shown in Figure 4.

The MCB image of the sSMC was similar to the MCB of the short arm of chromosome 12, indicating that the sSMC originated from 12p and their similar organization. Minor differences in the MCB images of the sSMC and 12p may be due to the absence in the sSMC of the proximal region of the long arm of chromosome 12, stained with PCP12-3, and additional rearrangements in the sSMC. To eliminate the possible influence on obtaining images of the sSMC and the short arm of chromosome 12, a two-color CISS-hybridization was performed with DNA probes PCP12-1 and PCP12-12, obtained from regions of the short arm of chromosome 12 (Supplementary Figure S4).

**FIGURE 5:**
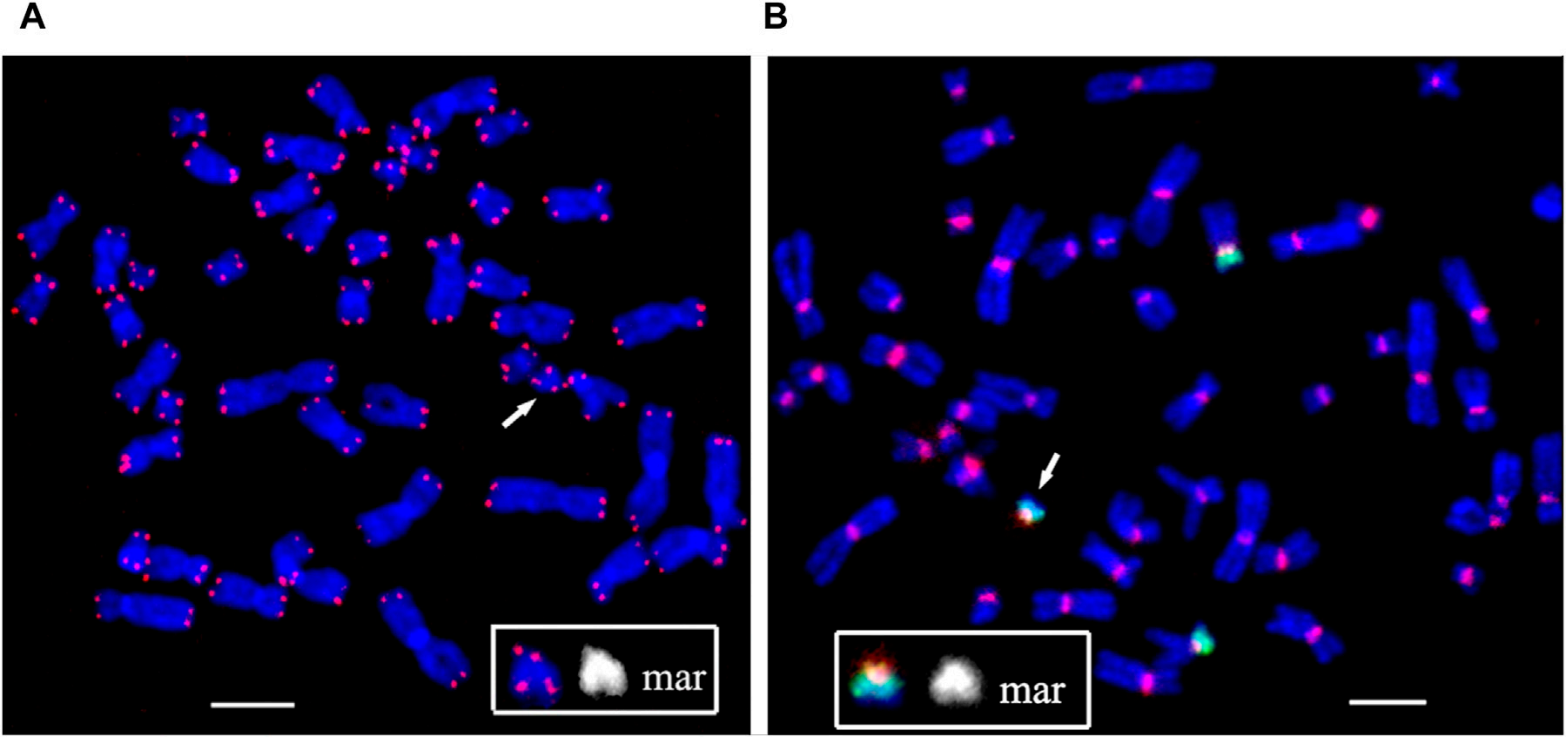
Fluorescence *in situ* hybridization of **(A)** the telomeric (red) and **(B)** the pan-centromeric (red) probes and PCP12-2 probe labeled with AlexaFluor488-5-dUTP (green) on metaphase chromosomes in peripheral blood lymphocytes of patient. The total number of chromosomes is 47. The arrow points to the small supernumerary marker chromosome 12 found in the patient’s karyotype. Scale bar 50 µm.

Usually, the sSMC in cases of Pallister-Killian Syndrome represents invdup12p, present in some cells, including a small percentage of peripheral blood cells (Dufke et al., 2001). However, in this patient, the sSMC was simply 12p, raising questions about the organization of its end at the deletion border. To solve this, FISH was performed with labeled telomeric and pan-centromeric DNA probes (Figure 5), as the morphology of the sSMC suggested a break in the pericentromeric region during its formation.

At the end of the sSMC formed as a result of the rearrangement, DNA characteristics of the pericentromeric regions of human chromosomes and a cluster of telomeric repeats were detected. That is, the sSMC is protected from degradation by telomeric repeats, and the presence of a centromeric region ensures normal mitotic separation. The second raises the question of the mechanism of mosaicism for the sSMC, characteristic of PKS.

Unfortunately, it must be acknowledged that even if there was a difference between the sSMC and the short arm of chromosome 12, the resolution level of the methods used did not allow it to be detected. For a higher-resolution analysis of the organization of the sSMC, aCGH was used in the study. Mosaicism for regions of interest can significantly complicate or make the use of this method practically impossible. However, the sSMC was detected in half of the peripheral blood cells, allowing for the successful use of aCGH. To confirm that the sSMC is present in a sufficient proportion of peripheral blood cells and that aCGH can be successfully used. A comparative quantitative assessment of the presence of DNA markers from the short arm of chromosome 12 in the patient’s peripheral blood cells was performed.

**FIGURE 6:**
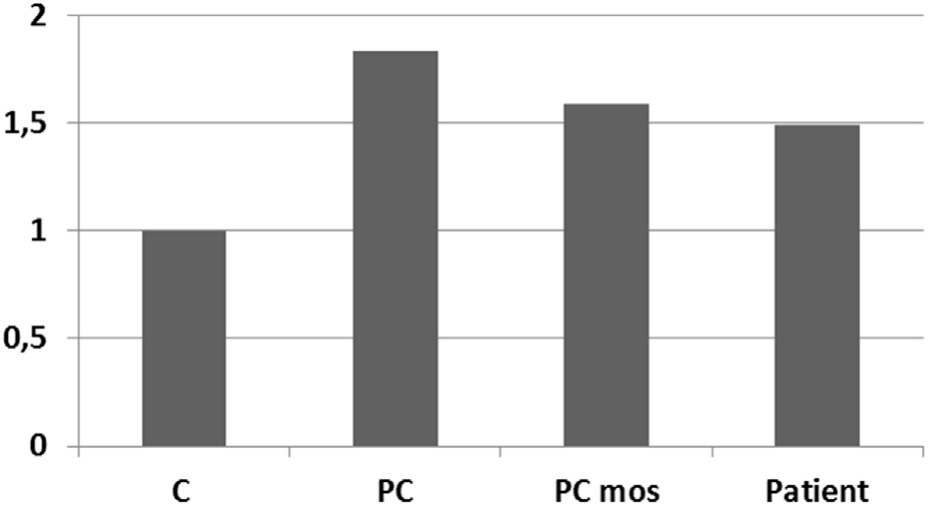
qPCR-validation of mosaicism level for sSMC(12) in a patient with PKS. Footnote. C–control DNA with normal karyotype (Agilent Technologies, United States); PC–positive control–DNA of a patient with dup12p13.31; PC mos–DNA of a patient with dup12p13.31 mixed with control DNA in a 1:1 ratio to model 50% mosaicism at the locus of interest; Patient - DNA of analyzed patient.

A comparative analysis of the results of real-time PCR of DNA extracted from the patient’s peripheral blood cells and control samples was performed: peripheral blood cells from a donor with a normal karyotype, a donor with a duplication in the 12p13.31 region (arr[hg19]12p13.31(8003414_808860)×3), a sample simulating the patient’s mosaicism (mixture of DNA from a healthy donor and a donor arr[hg19]12p13.31(8003414_808860)×3 in a 1:1 ratio). Results confirm the presence of 50% mosaicism for sSMC(12) in a patient (Figure 6). The study showed that the representation of the sSMC in peripheral blood cells is sufficient for aCGH. Despite the mosaicism detected in the patient, aCGH can be successfully used to analyze the DNA composition of the sSMC in peripheral blood cells for a more accurate description of the sSMC.

**FIGURE 7:**
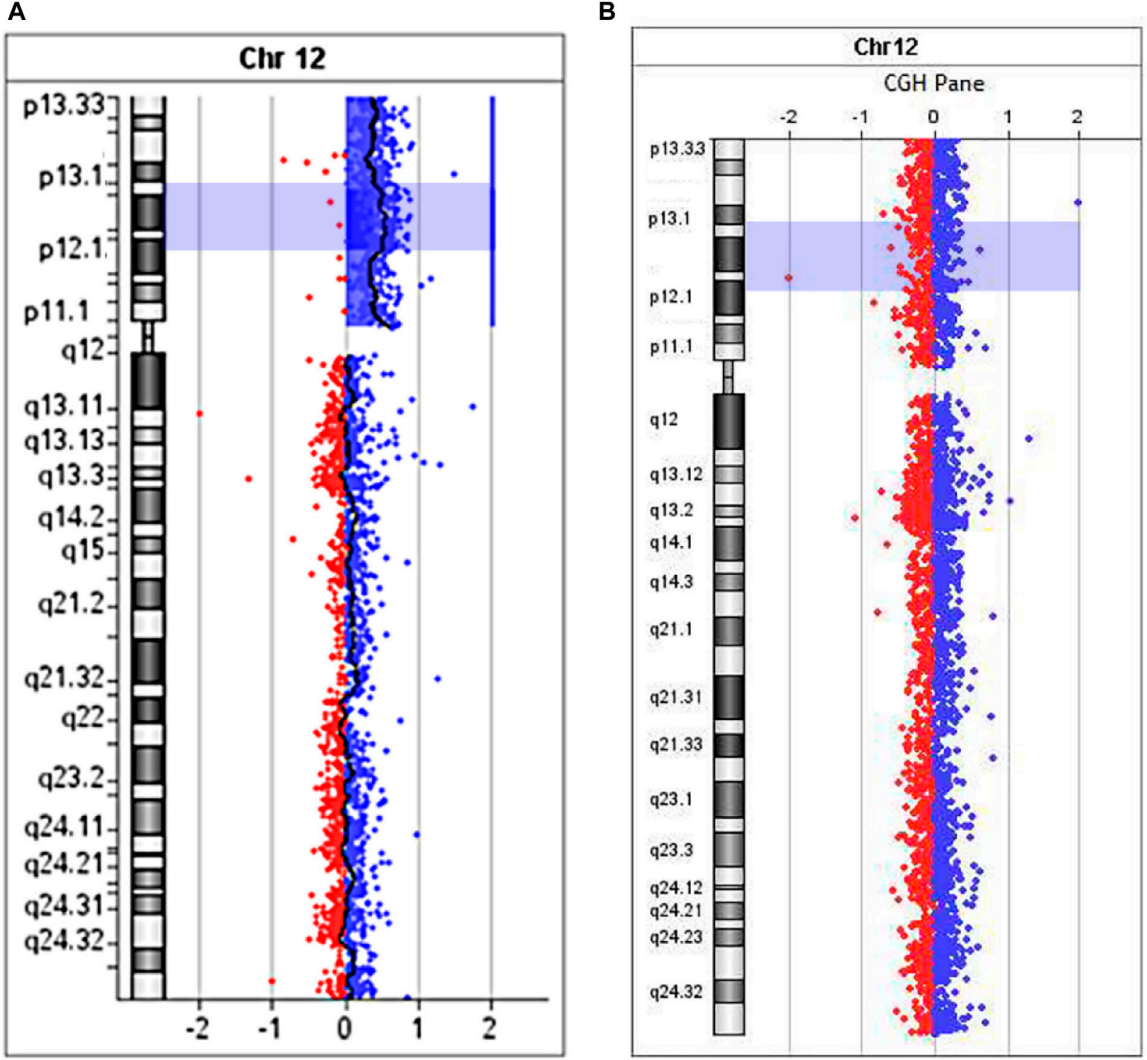
The aCGH profile of chromosome 12 with 12p13.33→p11.1 duplication for proband **(A)** and his mother **(B)**.

Microarray analysis (aCGH) was performed on SurePrint G3Human CGH 8 × 60K arrays (Agilent Technologies, United States) for proband (Figure 7A) and his mother (Figure 7B).

Maternal molecular karyotype revealed by aCGH was balanced (Figure 7B). As to the patient, the aCGH results revealed an excess in peripheral blood cells representation of the entire short arm of chromosome 12 DNA (12p13.33→p11.1, 34 Mb) (Figure 7A), which, along with the results of FISH investigations, unambiguously indicates the location of the breakpoint that took place during the formation of sSMC, in the pericentromeric heterochromatin region of the long arm of chromosome 12. Since no excess of DNA fraction in the distal region of the long arm of chromosome 12 was detected, it seems more probable that the sSMC was formed as a result of a break in the pericentromeric region and “repair” of the break by creating a telomeric repeat cluster by telomerase, leading to the loss of almost the entire long arm of chromosome 12. We did not obtain any data that the sSMC arose as a result of interstitial deletion, including almost the entire long arm. However, we cannot completely exclude that the loss of almost the entire long arm of chromosome 12 occurred as a result of interstitial deletion with one breakpoint in the pericentromeric region and the second either in the region of telomere-associated repeats or very close to them. It should be kept in mind that the rate of rearrangements involving subtelomeric regions is significantly increased (Veltman et al., 2002).

**FIGURE 8:**
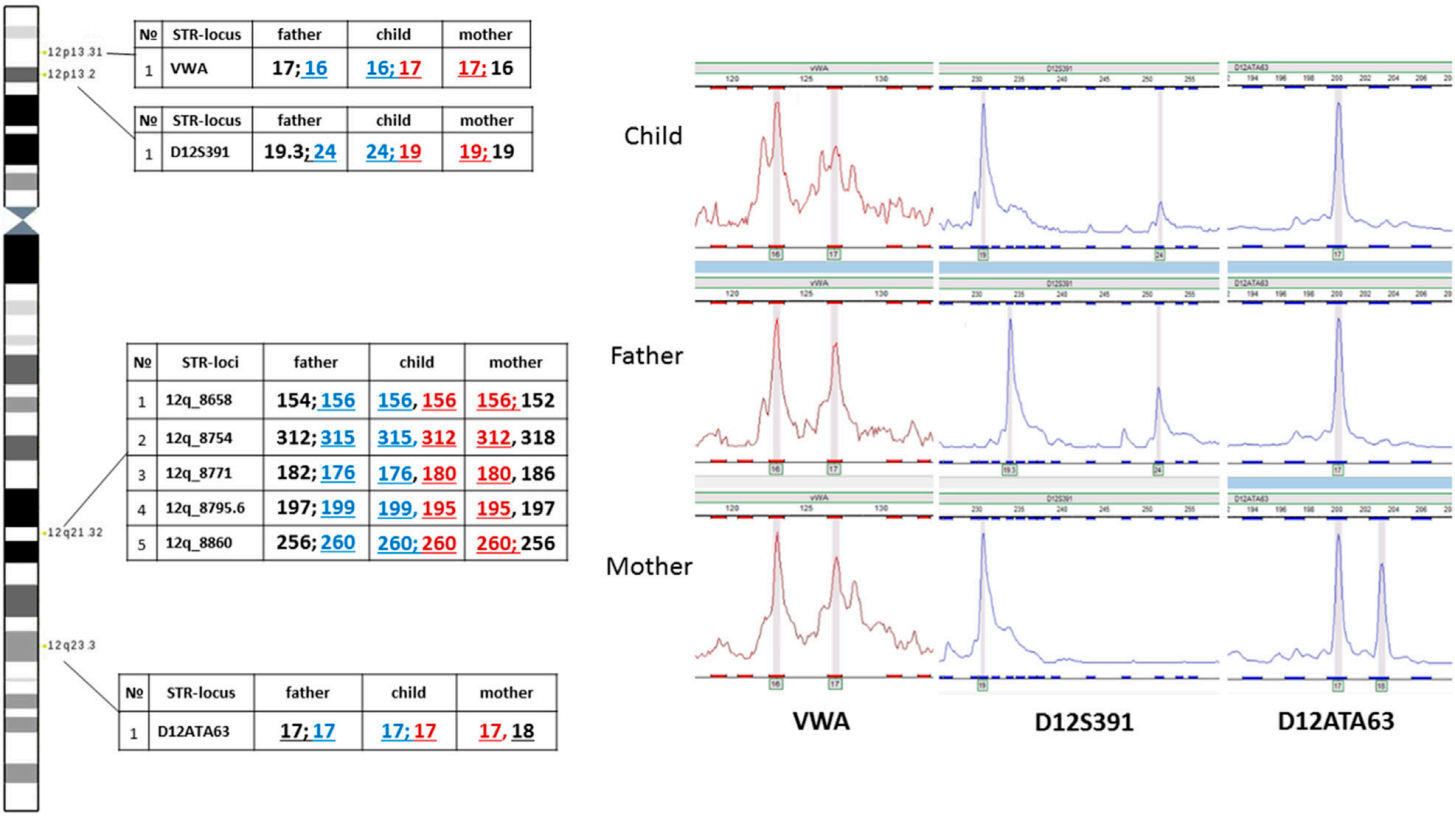
Familial analysis of chromosome 12 inheritance by STR genotyping.

The assumed mechanism of formation of the supernumerary chromosome allows for a chromosome non-disjunction event in meiosis with the emergence of a trisomic karyotype with subsequent correction of trisomy through the described hypothetical structural rearrangement of the supernumerary chromosome 12. In such a case, it does not rule out the possibility of uniparental disomy for chromosome 12 - UPD(12), which due to the localization on chromosome 12 of several genes with proven (*ST8SIA1, RBP5, HNF1A*) or assumed imprinted status (*ABCC9, SLC26A10, HOXC9, HOXC4, CDK4, FBRSL1, KIAA1545*) (Geneimprint, 2024, https://www.geneimprint.com/site/genes-by-species) may contribute additionally to the formation of clinical symptoms in patients. Moreover, the presence of UPD can lead to the homozygotization of recessive variants, which is also significant for forming a pathological phenotype. To test the assumption about the possible presence of UPD(12), we analyzed the inheritance of several polymorphic STR-loci located on the p and q arms of chromosome 12. No data indicating the presence of UPD(12) was obtained (Figure 8). In all informative cases, normal biparental inheritance of chromosome 12 was demonstrated, which, however, does not contradict the mechanism of trisomy correction, in which UPD arises only in 1/3 of cases.

Analysis of two polymorphic STR loci in the short arm of chromosome 12 was uninformative for determining the parental origin of the marker chromosome. On one hand, this could be explained by chromosomal mosaicism, which limits the sensitivity of STR analysis. On the other hand, visualization of normal biallelic inheritance of polymorphic loci in the short arm does not exclude the possibility of the emergence of the supernumerary chromosome either in meiosis II in one of the parents or at postzygotic stages of development.

Analyzing the results of aCGH, we noted a difference in the level of DNA excess in different regions of 12p. The excess of the DNA copy number level, pertaining to the region 12p13.1-p12.1 (8.956 Mb), is more significant than the DNA pertaining to other regions of 12p. Such differences cannot be caused by a partial duplication in the short arm of one of the copies of chromosome 12, as the excess of the DNA level corresponding to this region is less than what would be expected with the presence of such a duplication of the 12p13.1-p12.1 region in all cells of the patient. The data obtained when conducting aCGH can be explained by rearrangements of sSMC and the presence in the patient’s cells of different sSMCs or by the presence in the sSMC of a duplicated region corresponding to 12p13.1→p12.1. Mosaicism and the presence in peripheral blood cells of sSMCs with different deletions can mimic duplication of the region preserved in different variants of sSMCs.

**FIGURE 9:**
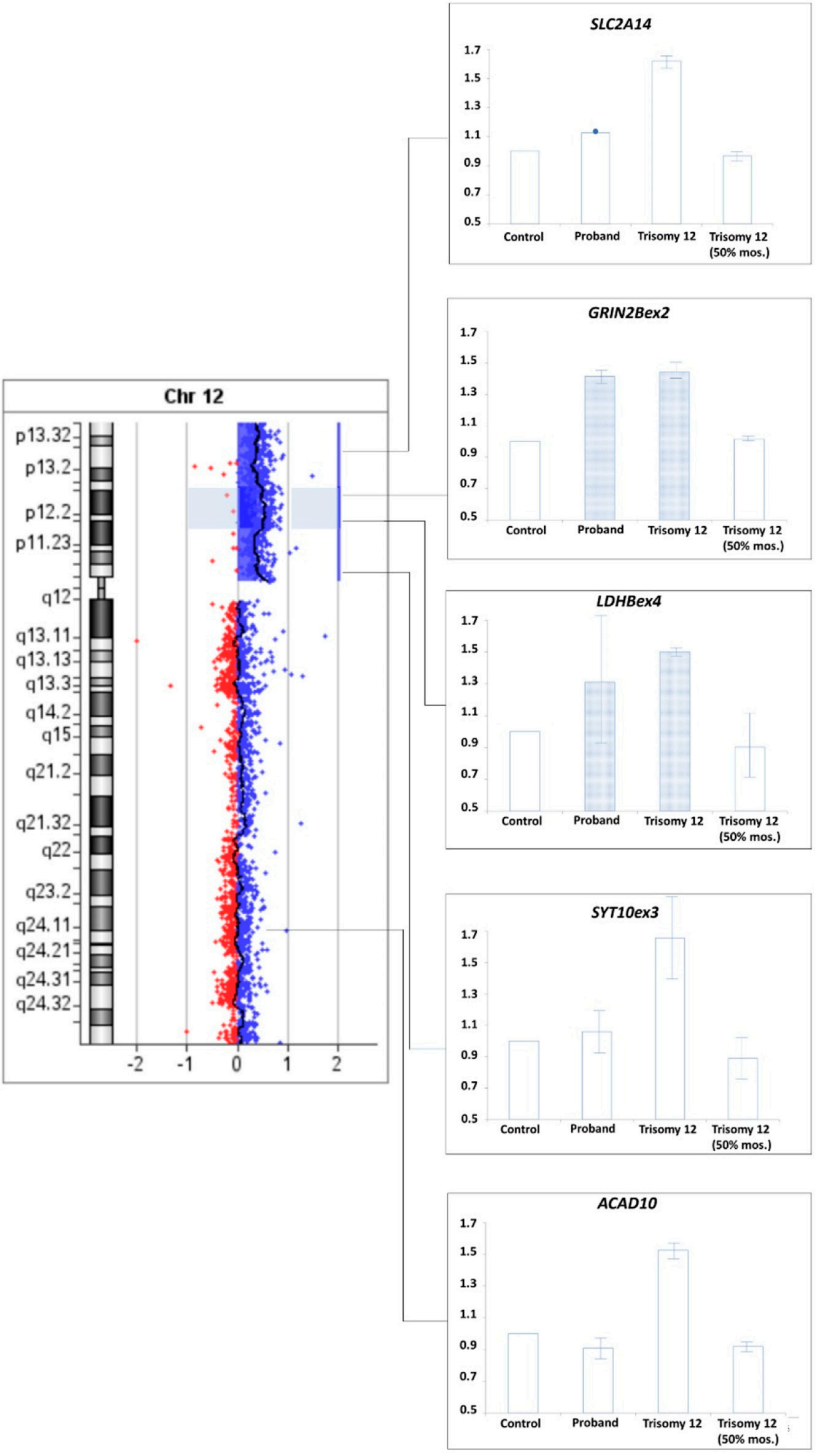
qPCR-validation of mosaicism level for sSMC(12) in a patient with Pallister-Killian syndrome. Footnote. Trisomy 12–DNA from cells of extraembryonic tissues of spontaneous abortion with trisomy 12, Triomy 12 (50% mosaicism)–DNA from cells of extraembryonic tissues of spontaneous abortion with trisomy 12 and control DNA from cells with normal karyotype in a 1:1 ratio.

To confirm this suggestion a comparative analysis of the results of real-time PCR of DNA isolated from the patient’s peripheral blood cells, control DNA, DNA from cells of extraembryonic tissues of spontaneous abortion with trisomy 12 (positive control 1) and DNA from a sample simulating mosaicism of the patient (a mixture of control DNA and DNA from cells of extraembryonic tissues of spontaneous abortion with trisomy 12 in a 1:1 ratio, positive control 2) was performed. The results for SLC2A4 and SYT10ex3 primers confirm at least 50% mosaicism for sSMC(12) in the patient. Results for GRIN2Bex2 and LDHBex4 primers indeed indicate the presence of partial tetrasomy in the 12p13.1→p12.1 region. Results for 12q24 region indicate a normal copy number of it in the patient (Figure 9).

**FIGURE 10:**
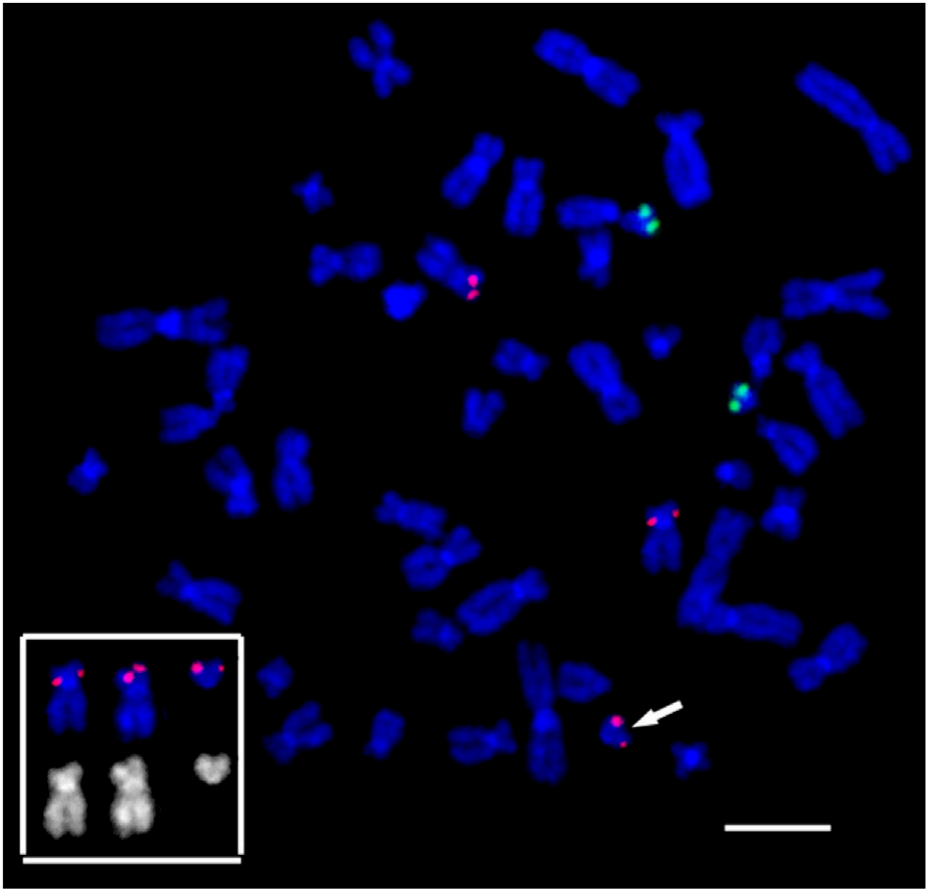
Suppression fluorescence *in situ* hybridization of locus-specific DNA probes on the patient’s metaphase chromosomes. FISH TEL/AML1 Translation, Dual Fusion Probe (Cytocell, Cambridge, UK). TEL (12p13.2)–red, AML1 (21q22.12)–green. Chromosome 12 and marker 12, bearing a red signal in the 12p13.2 locus, and their inverted DAPI banding are shown at the bottom left. The arrow points to the small supernumerary marker chromosome 12 found in the patient’s karyotype. General chromosome staining with DAPI (blue signal). Scale bar 50 µm.

At the same time, a lower level of signal excess in the band 12p13 than in 12p13.1→p12.1 allows us to suggest that if variations in the signal level when conducting aCGH are caused by the presence of different sSMCs with deletions in different regions, then among sSMCs there should be marker chromosomes that do not show a signal when conducting FISH with a locus-specific probe, marking the band 12p13. To check the alternative hypothesis and search for such marker chromosomes on the patient’s chromosomes, FISH was conducted with a commercial probe TEL/AML1 (Figure 10). FISH with TEL/AML1 marked subband 12p13.2 (red signal) and subband 21q22.12 (green signal). All marker chromosomes involved in the analysis (20 metaphase plates), like the short arms of chromosome 12, carried a red signal of the TEL/AML1 probe, which allowed us to conclude about the absence in the patient’s cells of sSMCs with a deletion in the band 12p13. Thus, the obtained data allowed us to reject the hypothesis about the presence in the sSMCs with deletion of the 12p13 band in the patient’s cells. They testify in favor of the hypothesis about the presence in sSMCs of a duplication corresponding to the region 12p13.31→p12.1 (13828759-22784758).

Summarizing the results of molecular cytogenetic and cytogenomic analysis, we can draw the next conclusions:

1. The sSMC, present in about half of peripheral blood cells, consists of material from the short arm of chromosome 12, centromere, pericentromeric region of the q-arm of chromosome 12, and a cluster of telomeric repeats, localized near the breakpoint in its pericentromeric region.
2. Information about mosaicism of the sSMC in cells of other tissues is missing, as consent for skin biopsy was not received.
3. The sSMC contains an 8.956 Mb interstitial duplication in the 12p13.1→p12.1 region that may be relevant to the formation of the patient’s clinical signs, corresponding to the PKS.
As a result of the performed studies, the patient’s karyotype was designated as 47,XY,+der(12).ishwcp(24XCyte)[10]/46,XY.ish wcp(24XCyte)[10].arr[hg19]12p13.33-p11.1(230421-34345585)×3[0.5]dn,12p13.1-p12.1(13828759-22784758)×4[0.5]dn.

## 4 Discussion

Performing genetic testing, we faced with the identification of copy number variation, which is associated with an imbalance of extended regions of the genome. The assessing the clinical significance of this changes is especially complicated since such changes affect a large number of genes, the structural organization of chromosomes, as well as the localization of genetic material in the interphase nucleus. Due to these changes in the spatial organization of the genome, the presence of an additional copy of chromosomal material may have a pathogenic effect, but sometimes have virtually no effect on the formation of the disease phenotype. For example, extra material present in a cell, in the form of a ring chromosome, may have less of an impact on development due to its localization in a transcriptionally inactive compartment of the nucleus (Lebedev et al., 2021). Cytogenetic diagnosis becomes even more complicated in the case of mosaicism, which is a hallmark for sSMC. One of the classic cases of mosaicism associated with a sSMC is invdup12p. Tetrasomy 12p leads to multiple developmental pathologies, the severity of which probably depends on what proportion of cells in specific tissues carry the marker chromosome. Unfortunately, this question remains open due to inaccessibility of different tissues for analysis. Available information is limited also to the low percentage of cells with invdup (12p) and their greater representation in the dividing skin fibroblasts. Significant differences in the proportion of cells with invdup (12p) may be due either to differences in the probability of differentiation of cells with and without invdup (12p) into different tissue types, or to different mortality in embryos with different variants of invdup (12p) mosaicism. We cannot even exclude that the frequency of conceptions with invdup (12p) may be very high in the early stages of development, but only variants with a certain type of mosaicism reach the later stages of embryogenesis and birth.

This study describes a patient with PKS whose cells carry a marker chromosome atypical for this syndrome and with its presence in an unusually high percentage of peripheral blood cells. It should be noted that sSMC is not just a short arm of chromosome 12, but also carries an interstitial *de novo* duplication of the chromosomal region 12p13.1→p12.1 with a size of 8.956 Mb. That is, cells carrying sSMC are characterized by trisomy of the short arm of chromosome 12, and tetrasomy in the 12p13.1→p12.1 region. It can be assumed that the birth of a child with a high proportion of sSMC in peripheral blood cells was possible due to the fact that most of the 12p material is presented in three rather than four copies.

**FIGURE 11:**
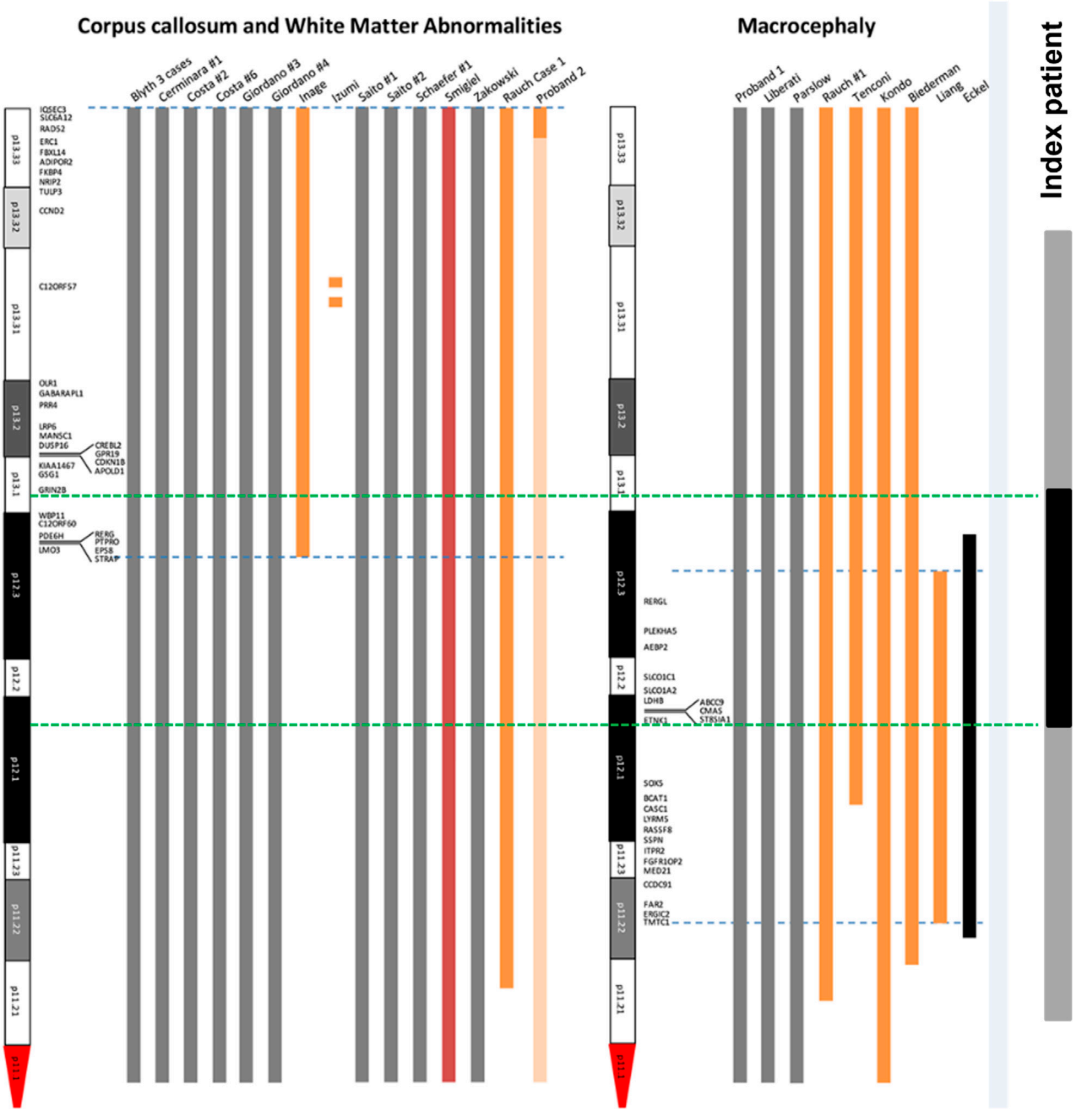
Mapping of candidate genes of structural brain abnormalities in patients with PKS. The figure is based and modified from the original map by Poulton et al. (2018) (Poulton et al., 2018). Blue dotted lines are the boundaries of localization of genes associated with abnormalities of corpus collosum and white matter (left) and macrocephaly (right). Green dotted lines are the boundaries of duplicated 12p13.1-p12.1 region (shown in black) within the sSMC(12) (shown in grey) in our patient.

In order to analyze the candidate genes of structural brain abnormalities in 12p13.1→p12.1 duplicated region in our patient we compared it with the minimal critical regions mapped by Poulton et al. for patients with PKS (Poulton et al., 2018) (Figure 11). It turned out that in our patient the 12p13.1→p12.1 duplicated region partially overlaps with both regions associated with abnormalities of the corpus callosum and white matter of the brain and macrocephaly. The common candidate genes are *WBP11, C12ORF60, PDE6H, RERG, PTPRO, EPS8, STRAP,* and *LMO3* for corpus collosum and white matter and *RERGL, PLEKHA5, AEBP2, SLCO1C1, SLCO1C2, LDHB, ABCC9, CMAS, ST8SIA1,* and *ETNK1* for macrocephaly. We should note that abnormalities of the mentioned brain structures and macrocephaly were detected in our patient during MRI.

In conclusion, our study is an example of a detailed description of a difficult-to-diagnose structural chromosomal abnormality, complicated by mosaicism of the patient’s karyotype. It is significant, that determining the clinical significance of unusual chromosome abnormalities for classical chromosomal syndrome, like PKS, requires application of different complementary molecular cytogenetic and cytogenomic techniques.

## Data Availability

The datasets for this article are not publicly available due to concerns regarding participant/patient anonymity. Requests to access the datasets should be directed to the corresponding author.
