# Supplementary material for: A case report of Pallister-Killian syndrome with an unusual mosaic supernumerary marker chromosome 12 with interstitial 12p13.1-p12.1 duplication
Source: Front Genet. 2024 Mar 11;15:1331066. doi: 10.3389/fgene.2024.1331066 (PMC10961358; doi:10.3389/fgene.2024.1331066)
Supplement: Supplementary file 1 [file DataSheet1.zip › Data Sheet 1/Data Sheet 1.docx]

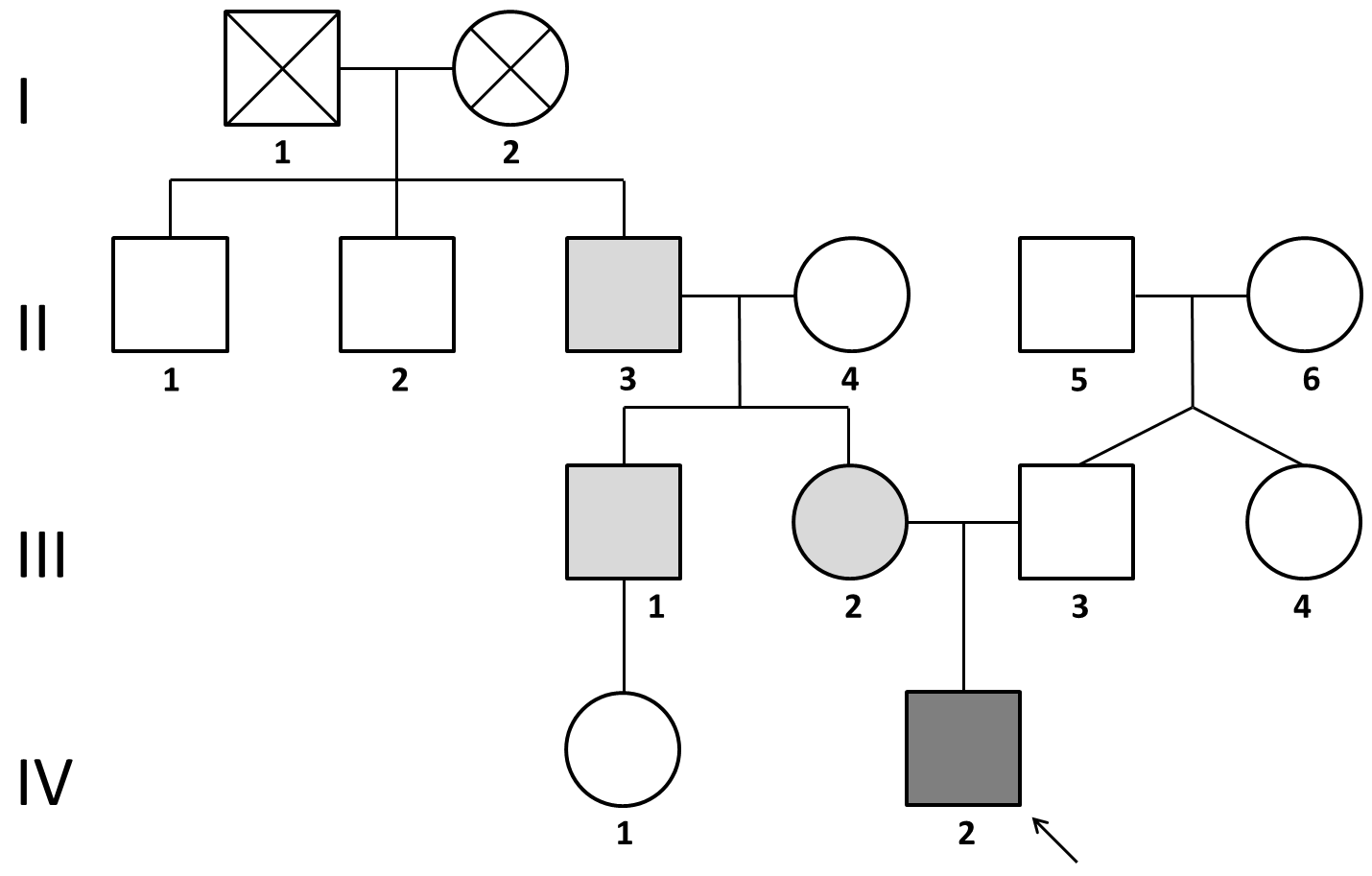


**Figure S1.** **The pedigree of the family. II,** 3 – preaxial polydactyly of the feet, syndactyly of I and II toes; **III**, 1 – syndactyly of the III and IV fingers of the left hand; **III**, 2 – syndactyly of the III and IV fingers of the left hand, phenotype features; **IV**, 2 – proband with PKS diagnosis. Only family members with symptoms are commented.

| **A** | **B** |
| --- | --- |
| 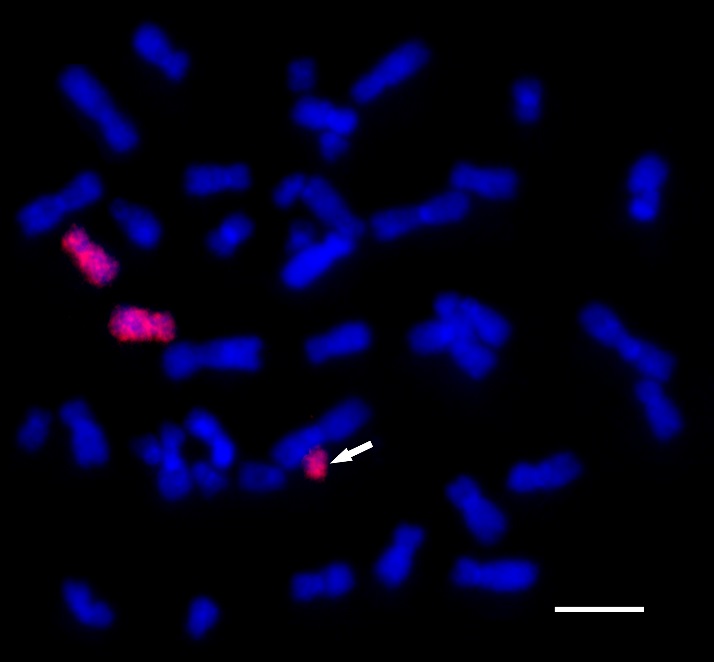 | 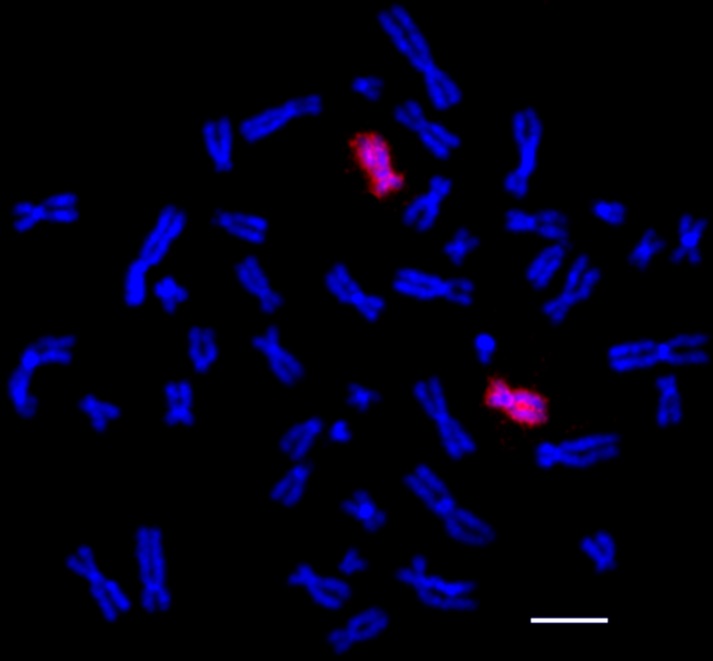 |

**Figure S2. Suppression fluorescence *in situ* hybridization WCP12 with the patient's metaphase chromosomes.** (**A**) A metaphase plate with a marker chromosome. (**B**) A metaphase plate without a marker chromosome. The WCP12 DNA probe signal is red. DAPI chromosome staining is blue. The arrow points to the small supernumerary marker chromosome 12 found in the patient's karyotype. Scale bar 50 µm.

| **A** | **B** |
| --- | --- |
| 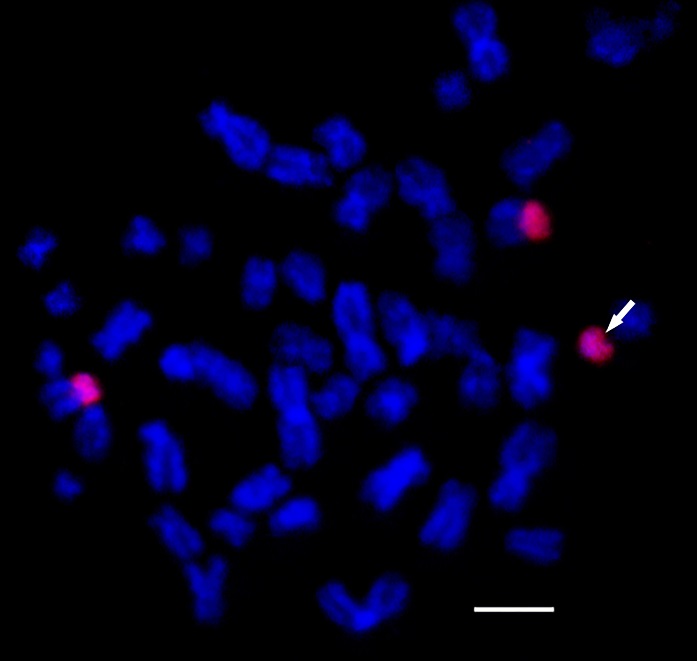 | 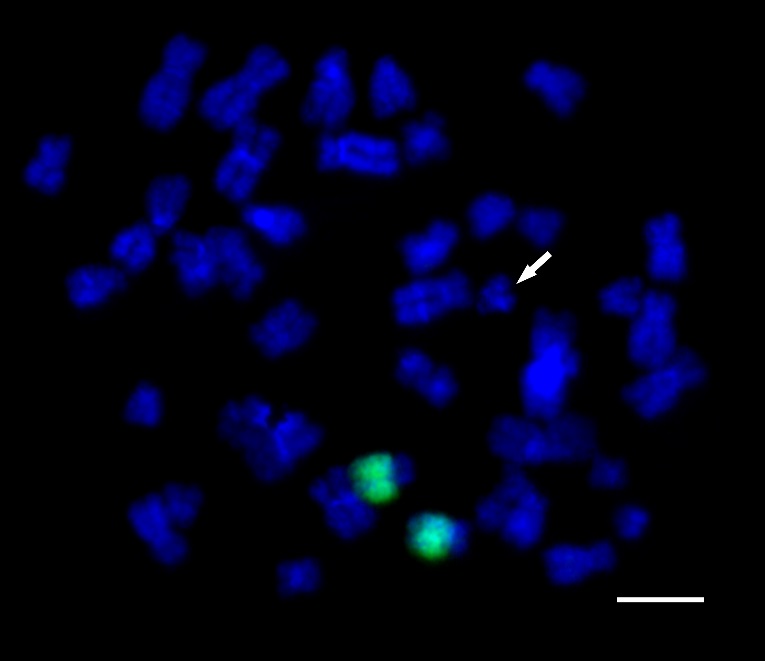 |

**Figure S3. Suppression fluorescence *in situ* hybridization of arm-specific DNA probes on the patient's metaphase chromosomes.** (**A**) The FISH signal of the PCP12p DNA probe is red. (**B**) FISH signal of the PCP12q DNA probe is green. General chromosome staining with DAPI (blue signal). The arrow points to the small supernumerary marker chromosome 12 found in the patient's karyotype with (A) and without (B) hybridization signal. Scale bar 50 µm.


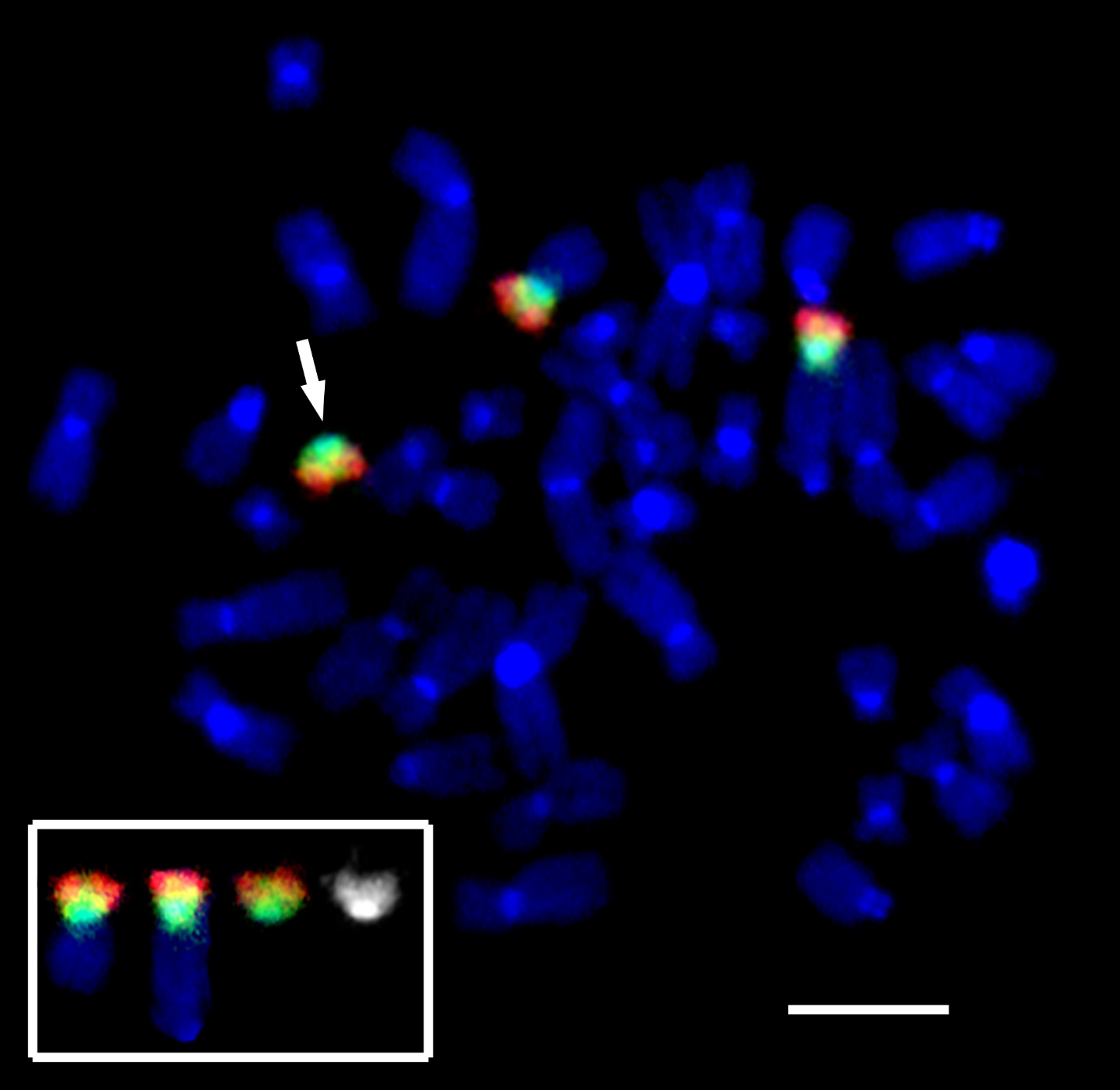


**Figure S4. Suppression fluorescence *in situ* hybridization of locus-specific DNA probes on the patient's metaphase chromosomes.** The FISH signal of the PCP12-1 DNA probe is red; the FISH signal of the PCP12p-2 DNA probe is green. At the bottom left are the chromosomes on which a specific signal was detected and the inverted DAPI banding of the marker chromosome. The arrow points to the small supernumerary marker chromosome 12 found in the patient's karyotype. General chromosome staining with DAPI (blue signal). Scale bar 50 µm.

**TABLE S1 Primers used for real-time PCR.**

| Region | Primer | Sequence |
| --- | --- | --- |
| 5q13.3 | HEXB F | 5′-CCGGGCACAATAGTTGAAGT-3′ |
|  | HEXB R | 5′-TCCTCCAATCTTGTCCATAGC-3′ |
| 12p13.33-p11.1 | SLC2A14 F | 5'-TTGTGAGGAAGTGTCTTTTGGC-3' |
|  | SLC2A14 R | 5'-TGGTAGGTATCTTTCAAGGCCA-3' |
|  | SYT10ex3 F | 5’-TTTTGACAGATTTTCTAGACATGACA-3’ |
|  | SYT10ex3 R | 5’-GCTTCCCTGGAGAGATCAGA-3’ |
| 12p13.1-p12.1 | GRIN2Bex2 F | 5'-GAGACCGACCCAAAGAGCA-3' |
|  | GRIN2Bex2 R | 5'-TGTGTCATCAGCAAACACCAC-3' |
|  | LDHBex4 F | 5'-TTCTGTGACCGCCAATTCTAAG-3' |
|  | LDHBex4 R | 5'-CCAGATTGAGCCGACTCTCC-3' |
| 12q24 | ACAD10 F | 5’-GAAGCCTGGAGTCTGTTTCAG-3’ |
|  | ACAD10 R | 5’-CATTTCTGTATGGTCAGCACCT-3’ |

**Table S2 Characteristics of STR loci included in the study.**

| № | Region | STR-loci | Repeat | Heterozygosity | Size, bp |
| --- | --- | --- | --- | --- | --- |
| 1 | 12p13.31 | VWA | TCTA[TCTG]_4_[TCTA]_13_ | 0.8 | 85-157 |
| 2 | 12p13.2 | D12S391 | [AGAT]_5_ GAT[AGAT]_7_ [AGAC]_6_AGAT | 0.8 | 203-267 |
| 3 | 12q21.32 | 12q_8658 | AC | 0.8 | 150-164 |
| 4 |  | 12q_8754 | AAT | 0.8 | 310-322 |
| 5 |  | 12q_8771 | AC | 0.8 | 170-188 |
| 6 |  | 12q_8795.6 | AT | 0.8 | 195-210 |
| 7 |  | 12q_8860 | AAGG | 0.7 | 250-270 |
| 8 | 12q23.31 | D12ATA63 | [TAA][CAA] | 0.8 | 76–106 |
