# Supplementary material for: A case report of Pallister-Killian syndrome with an unusual mosaic supernumerary marker chromosome 12 with interstitial 12p13.1-p12.1 duplication
Source: Front Genet. 2024 Mar 11;15:1331066. doi: 10.3389/fgene.2024.1331066 (PMC10961358; doi:10.3389/fgene.2024.1331066)
Supplement: Supplementary file 1 [file DataSheet1.zip › Data Sheet 1/Table S1.docx]

**TABLE S1 Primers used for real-time PCR.**

| Region | Primer | Sequence |
| --- | --- | --- |
| 5q13.3 | HEXB F | 5′-CCGGGCACAATAGTTGAAGT-3′ |
|  | HEXB R | 5′-TCCTCCAATCTTGTCCATAGC-3′ |
| 12p13.33-p11.1 | SLC2A14 F | 5'-TTGTGAGGAAGTGTCTTTTGGC-3' |
|  | SLC2A14 R | 5'-TGGTAGGTATCTTTCAAGGCCA-3' |
|  | SYT10ex3 F | 5’-TTTTGACAGATTTTCTAGACATGACA-3’ |
|  | SYT10ex3 R | 5’-GCTTCCCTGGAGAGATCAGA-3’ |
| 12p13.1-p12.1 | GRIN2Bex2 F | 5'-GAGACCGACCCAAAGAGCA-3' |
|  | GRIN2Bex2 R | 5'-TGTGTCATCAGCAAACACCAC-3' |
|  | LDHBex4 F | 5'-TTCTGTGACCGCCAATTCTAAG-3' |
|  | LDHBex4 R | 5'-CCAGATTGAGCCGACTCTCC-3' |
| 12q24 | ACAD10 F | 5’-GAAGCCTGGAGTCTGTTTCAG-3’ |
|  | ACAD10 R | 5’-CATTTCTGTATGGTCAGCACCT-3’ |
