# Supplementary material for: A case report of Pallister-Killian syndrome with an unusual mosaic supernumerary marker chromosome 12 with interstitial 12p13.1-p12.1 duplication
Source: Front Genet. 2024 Mar 11;15:1331066. doi: 10.3389/fgene.2024.1331066 (PMC10961358; doi:10.3389/fgene.2024.1331066)
Supplement: Supplementary file 1 [file DataSheet1.zip › Data Sheet 1/Table S2.docx]

## TABLE S2 Characteristics of STR loci included in the study.

| № | Region | STR-loci | Repeat | Heterozygosity | Size, bp |
| --- | --- | --- | --- | --- | --- |
| 1 | 12p13.31 | VWA | TCTA[TCTG]_4_[TCTA]_13_ | 0.8 | 85-157 |
| 2 | 12p13.2 | D12S391 | [AGAT]_5_ GAT[AGAT]_7_ [AGAC]_6_AGAT | 0.8 | 203-267 |
| 3 | 12q21.32 | 12q_8658 | AC | 0.8 | 150-164 |
| 4 |  | 12q_8754 | AAT | 0.8 | 310-322 |
| 5 |  | 12q_8771 | AC | 0.8 | 170-188 |
| 6 |  | 12q_8795.6 | AT | 0.8 | 195-210 |
| 7 |  | 12q_8860 | AAGG | 0.7 | 250-270 |
| 8 | 12q23.31 | D12ATA63 | [TAA][CAA] | 0.8 | 76–106 |
