# Supplementary figures and images for: A case report of Pallister-Killian syndrome with an unusual mosaic supernumerary marker chromosome 12 with interstitial 12p13.1-p12.1 duplication
Source: Front Genet. 2024 Mar 11;15:1331066. doi: 10.3389/fgene.2024.1331066 (PMC10961358; doi:10.3389/fgene.2024.1331066)

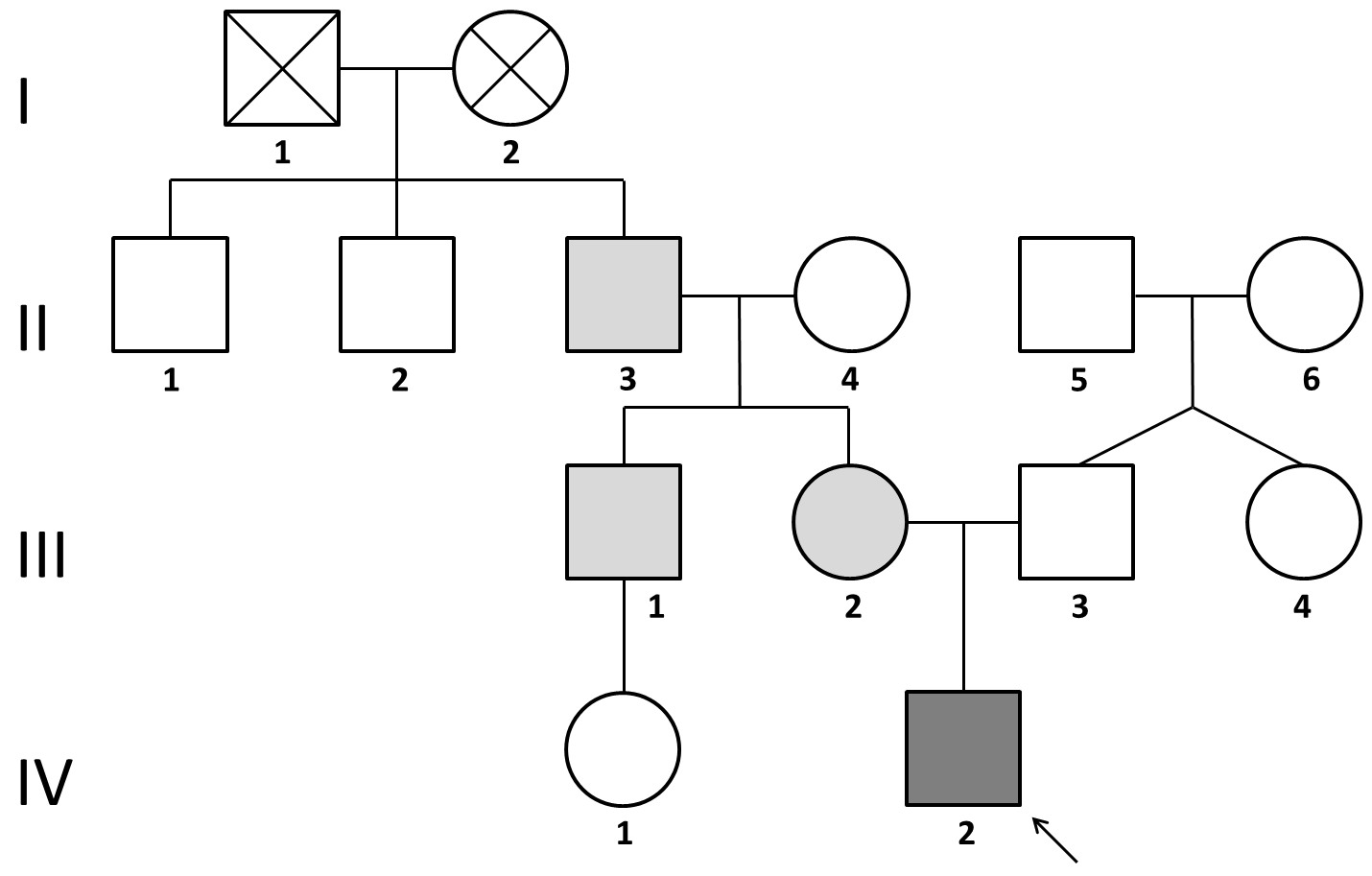

Supplement: Supplementary file 1 [file DataSheet1.zip › Data Sheet 1/Figure S1.tif]

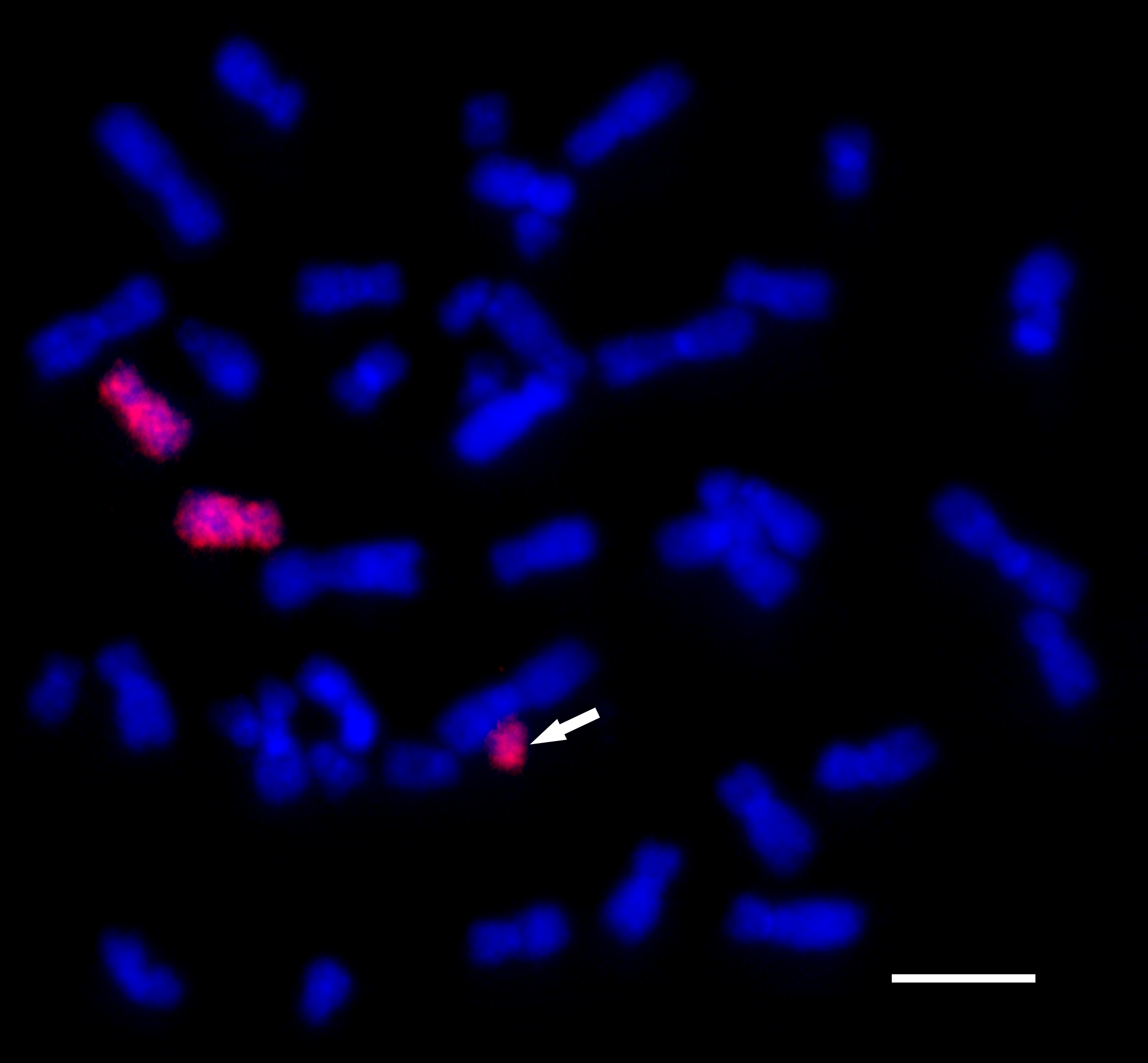

Supplement: Supplementary file 1 [file DataSheet1.zip › Data Sheet 1/Figure S2A.jpg]

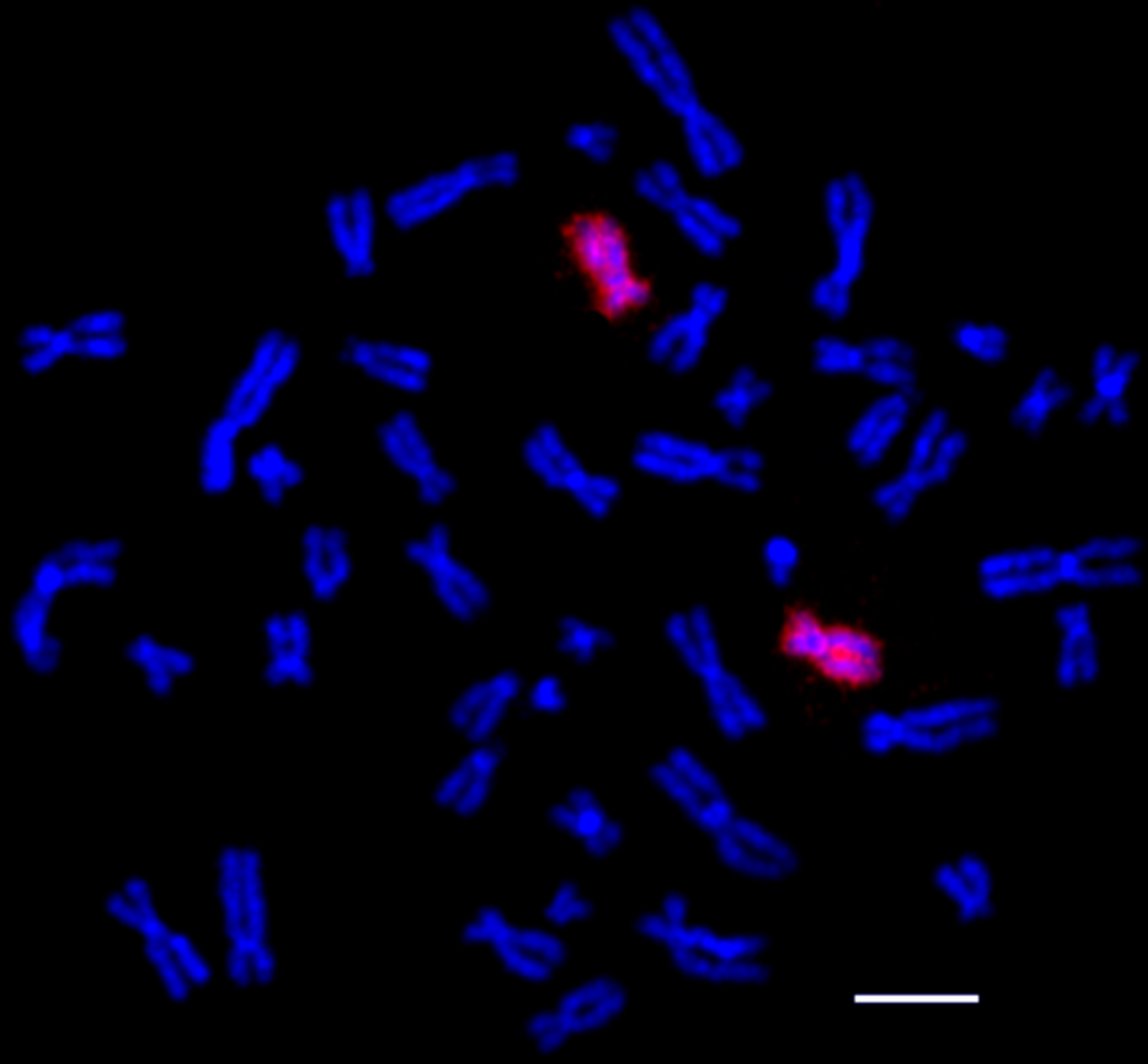

Supplement: Supplementary file 1 [file DataSheet1.zip › Data Sheet 1/Figure S2B.jpg]

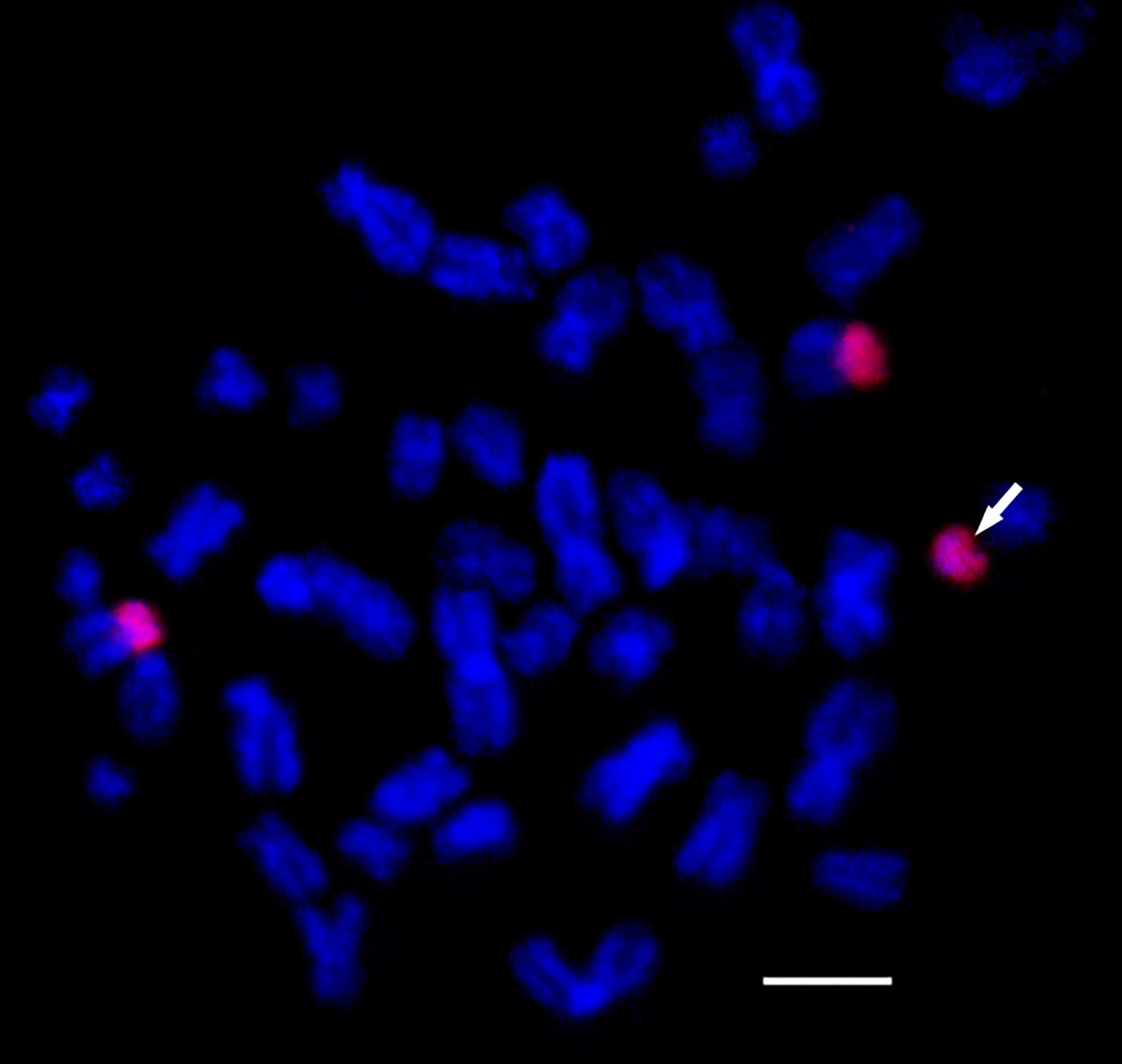

Supplement: Supplementary file 1 [file DataSheet1.zip › Data Sheet 1/Figure S3A.jpg]

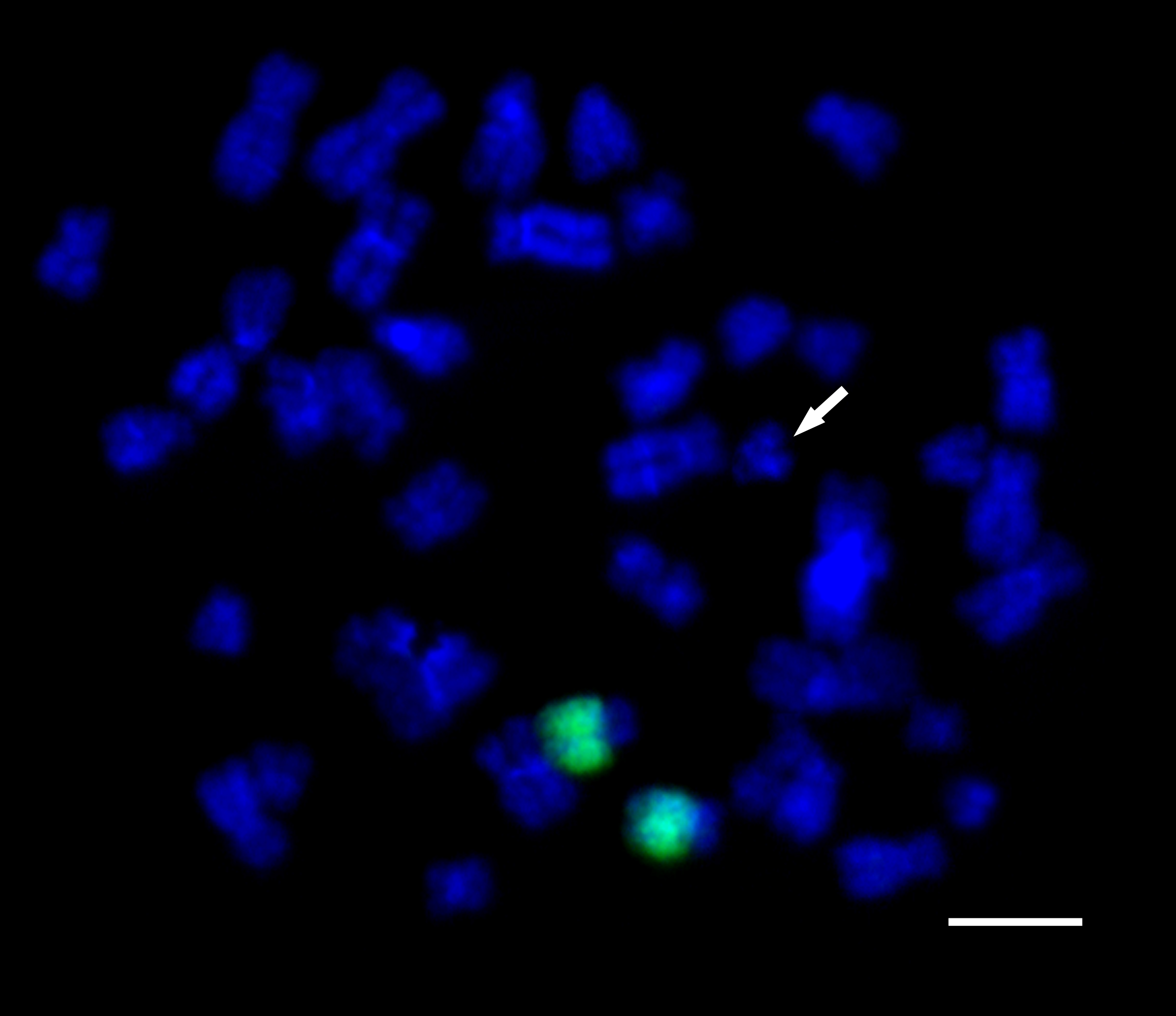

Supplement: Supplementary file 1 [file DataSheet1.zip › Data Sheet 1/Figure S3B.jpg]

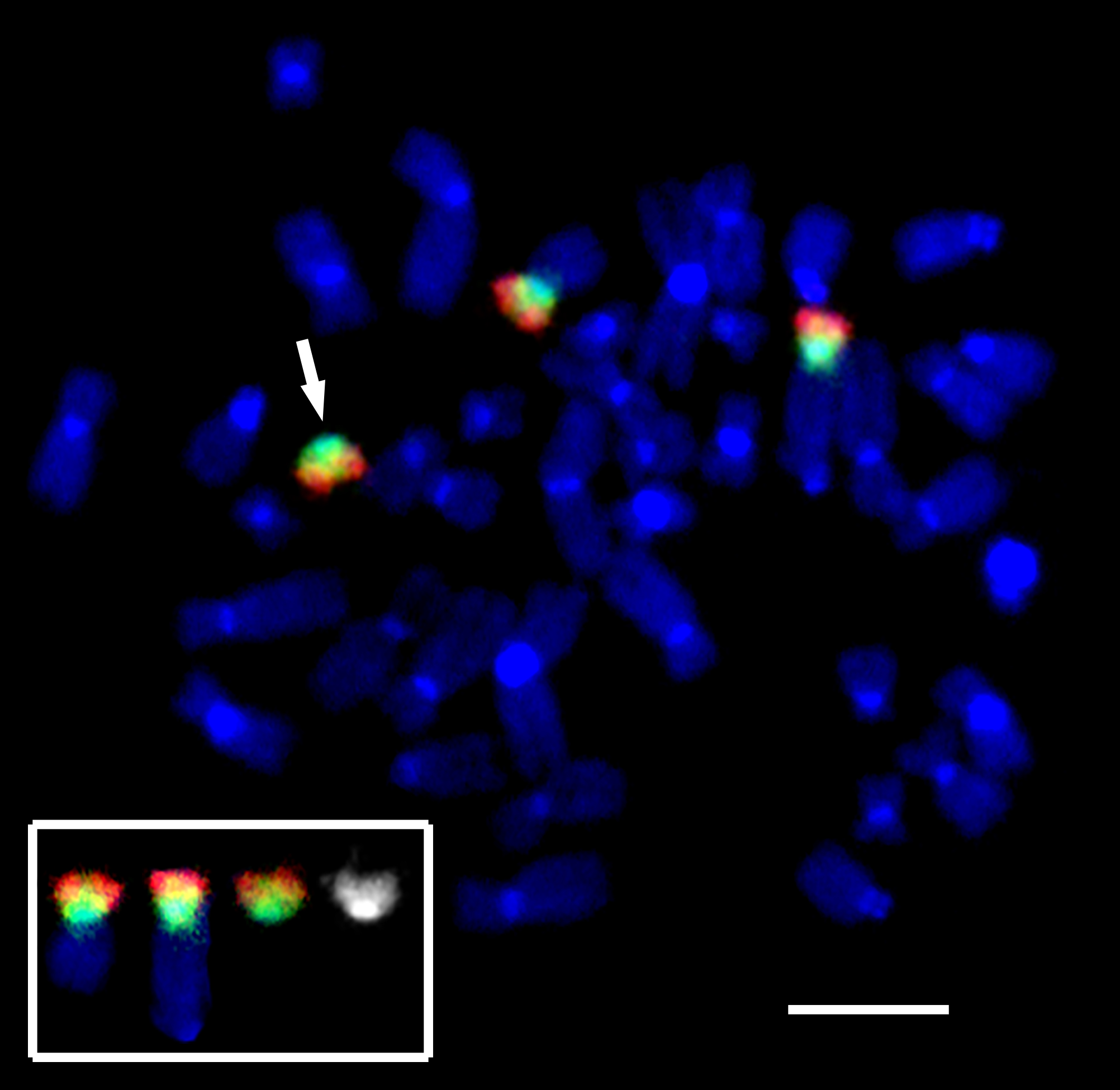

Supplement: Supplementary file 1 [file DataSheet1.zip › Data Sheet 1/Figure S4.jpg]
